# Deciphering the Impact of Mutations on *Pf*DHPS Active Site and Sulfadoxine Binding: Structural Insights from Molecular Dynamics Simulations

**DOI:** 10.3390/molecules30204118

**Published:** 2025-10-17

**Authors:** Emilie Guémas, Sandie Ménard, Nicolas Jeanne, Georges Landa, Antoine Berry, Marie Brut

**Affiliations:** 1 Laboratoire d’Analyse et d’Architecture des Systèmes (LAAS)-CNRS, Université de Toulouse, CNRS, 31031 Toulouse, France; jeanne.n@chu-toulouse.fr (N.J.); georges.landa@laas.fr (G.L.); marie.brut@laas.fr (M.B.); 2 Institut Toulousain des Maladies Infectieuses et Inflammatoires (Infinity), Université de Toulouse, CNRS UMR 5051, INSERM UMR 1291, 31024 Toulouse, France; sandie.menard@inserm.fr (S.M.); berry.a@chu-toulouse.fr (A.B.); 3 Service de Parasitologie et Mycologie, Centre Hospitalo-Universitaire (CHU) Toulouse, 31300 Toulouse, France

**Keywords:** dhps, sulfadoxine, *Plasmodium falciparum*, drug resistance, molecular dynamic simulations

## Abstract

The antimalarial combination of sulfadoxine–pyrimethamine is used as a preventive treatment in pregnant women and children in Africa. Sulfadoxine inhibits the *Plasmodium falciparum* dihydropteroate synthase (*Pf*DHPS), but resistance has emerged through point mutations in this enzyme. In this study, we investigate the impact of mutations on the structural and dynamic properties of *Pf*DHPS using molecular dynamics simulations. Our results show that *Pf*DHPS maintains overall structural integrity across various combinations of resistance-associated mutations. However, significant differences emerge in ligand binding. Sulfadoxine binding is particularly impacted and shows reduced stability in the mutant systems compared to the wild-type enzyme, while the natural substrate generally maintains stable or even enhanced binding affinity. A key finding is the critical role of the D2 loop, whose conformational flexibility influences ligand retention. In mutant enzymes, the disruption of interactions between the D2 loop and the natural substrate correlates with decreased affinity. In contrast, specific mutations in the loop are associated with an increased affinity. Conversely, sulfadoxine binding is associated with an open D2 loop conformation, facilitating its release from the active site. Finally, the intrinsic flexibility of sulfadoxine emerges as an important determinant of this process. Together, these results provide molecular-level insights into the mechanisms of resistance in *Pf*DHPS and establish a structural and dynamic framework for future investigations into its catalytic function and inhibitor design.

## 1. Introduction

Malaria remains a devastating infectious disease, with high morbidity and mortality among pregnant women and children. The antimalarial sulfadoxine–pyrimethamine (SP) combination is used for intermittent preventive treatment in pregnant women throughout Africa. Moreover, seasonal malaria chemoprevention with SP plus amodiaquine has been implemented in nineteen countries in sub-Saharan Africa for children under five years of age [1].

**Figure 1:**
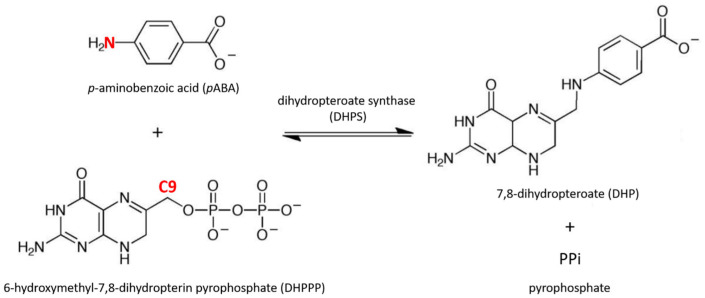
Reaction catalyzed by dihydropteroate synthase. DHPS facilitates the condensation of 6-hydroxymethyl-7,8-dihydropterin pyrophosphate (DHPPP) with *p*-aminobenzoic acid (*p*ABA) to form 7,8-dihydropteroate (DHP) and releases pyrophosphate. During the reaction, a covalent bond forms between the N atom of *p*ABA and C9 atom of DHPPP (highlighted in red).

Sulfadoxine (SDX) and pyrimethamine act synergistically on the obligate folate biosynthesis pathway of *Plasmodium falciparum* parasite, by inhibiting dihydropteroate synthase (DHPS) and dihydrofolate reductase (DHFR), respectively. *Plasmodium falciparum* DHPS is fused to 6-hydroxymethyl-7,8-dihydropterin pyrophosphokinase (HPPK) to form HPPK-DHPS, a bifunctional enzyme that catalyzes sequential reactions in the folate biosynthesis pathway. HPPK catalyzes the transfer of a pyrophosphate group from ATP to 6-hydroxymethyl-7,8-dihydropterin to form 6-hydroxymethyl-7,8-dihydropterin pyrophosphate (DHPPP). Then, DHPS catalyzes the condensation of DHPPP to *p*-aminobenzoic acid (*p*ABA) to form 7,8-dihydropteroate (DHP) as a substrate for folate synthesis (Figure 1) [2]. SDX competes with *p*ABA by mimicry, inhibiting DHP synthesis and thus reducing the synthesis of tetrahydrofolate, essential, in particular, for parasite DNA synthesis.

**Figure 2:**
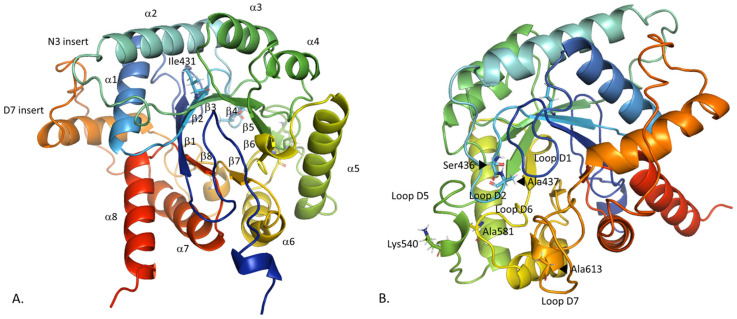
Annotated view of the Wild-Type *Plasmodium falciparum* dihydropteroate synthase. The view towards the barrel (**A**) and rotate 90° along the vertical axis (**B**). The six main point mutations are represented in (**B**) except the Ile431 for clarity. Ile431 is located in the β2-strand, Ser436 and Ala437 in the loop D2, Lys540 in the loop D5, Ala581 in the loop D6 and Ala613 in the loop D7. Loops D1 (residues 397–410), D2 (433–444), D3 (483–485), D4 (504–510), D5 (530–547), D6 (576–583), D7 (605–619) and D8 (687–689). β1-strand (residues 390–396), β2-strand (428–432), β3-strand (478–482), β4-strand (500–503), β5-strand (525–529), β6-strand (572–575), β7-strand (602–604) and β8-strand (684–687). α1-helice (411–424), α2-helice (445–461), α3-helice (486–494), α4-helice (511–518), α5-helice (549–565), α6-helice (584–597), α7-helice (664–680) and α8-helice (690–704). Insert N3 (466–472), insert D7 (620–660).

*Plasmodium falciparum* has developed resistance to SDX due to point mutations in *Pf*DHPS. So far, six main point mutations (Ile431Val, Ser436Ala, Ala437Gly, Lys540Glu, Ala581Gly, Ala613Ser) have been reported, with uneven frequencies across the African continent. These mutations are located within four flexible and conserved loops (Ser436 and Ala437 in the loop D2 (residues 433–444), Lys 540 in the loop D5 (530–547), Ala581 in the loop D6 (576–583) and Ala613 in the loop D7 (605–619)), with the exception of Ile431 located in the β2-strand (residues 428–432) (Figure 2) [3]. Ile431 is the last described point mutation, found in 2007 in Nigeria [4], then in Cameroon in 2010 [5]. The quintuple mutant vagKgs (Ile431Val, Ser436Ala, Ala437Gly, Ala581Gly and Ala613Ser) is the most common allele carrying this mutation (*Pf*dhps alleles are named according to the amino acid present at positions 431, 436, 437, 540, 581, and 613; the wild-type (WT) amino acid is stated in uppercase, and the mutated amino acid in lowercase). These mutations are being monitored as they have emerged in West Africa, without any clinical evidence of resistance [6]. The triple mutant ISgegA (Ala437Gly, Lys540Glu and Ala581Gly) is associated with a high level of resistance and is found with a high prevalence in Eastern and Southern Africa [7]. The combination of Lys540Glu and Ile431Val mutations has not been observed in any published data [6].

In this study, we investigate the effects of mutations on the structural and dynamic properties of the *Pf*DHPS enzyme. Prior to the availability of the crystal structure, molecular dynamics (MD) had already been used to address SDX resistance, but relying on homology models [8,9]. Here, we worked on the *Pf*DHPS crystal structure, first released in 2020 [3], using it as a starting model that we completed, and conducted extensive MD simulations to assess the impact of key mutations on the protein structure, as well as its binding to both the natural substrate (*p*ABA) and its inhibitor (SDX). Multiple combinations of *Pf*DHPS mutations, guided by epidemiological data, have been systematically investigated. Particular attention is given to the Ile431Val mutation, with the aim of gaining deeper mechanistic insight into the molecular basis of SDX resistance in *P. falciparum*.

## 2. Results

### 2.1. Comparison of the Constructed PfDHPS Structure with the AlphaFold2 Prediction

The crystal structure of *Pf*DHPS was recently published, but with missing parts [3]. In this work, the initial structure of WT *Pf*DHPS obtained by filling in the missing parts of the PDB entry 6JWQ (83 residues) with DHPS from *P. falciparum* and *P. vivax*, was compared with the structure predicted by AlphaFold2 [10]. The predicted local distance difference test (pLDDT) of the *Pf*DHPS structure obtained with AlphaFold2 corresponds to high confidence (87.739). In both cases, we identify the DHPS classical triosephosphate isomerase (TIM) barrel α/β structure with eight α-helices and eight β-strands (Figure 2). The primary structural difference between the two models lies in the insert D7 (residues 620–660). Structural alignment provides a RMSD of 0.260 Å over 252 retained residues, indicating strong agreement between the models (Figure S1). Most of the excluded residues correspond to insert D7, which is not solved in experimental structures and is poorly defined in the AlphaFold2 prediction. Moreover, this insert is assumed to be involved and stabilized at the *Pf*HPPK-DHPS dimer interface [3]. Accordingly, we consider the observed conformational variability in insert D7 to be acceptable in the monomeric form and sufficiently distant from the active site not to affect our conclusions. In the following part, our results support this assumption.

### 2.2. Compared Dynamics Properties of the Wild-Type and Mutated PfDHPS in Complex with pABA

As expected, the total energy of the eight systems is constant during the 200 ns production runs, ensuring the simulations are stable (Figure S2). RMSD values were computed along the trajectories both including and excluding the insert D7 region (Figure S3). When all *Pf*DHPS residues are considered, average RMSD values range from 3.58 ± 0.63 Å (vagKgs) to 6.20 ± 0.69 Å (WT) (Table 1). Upon exclusion of insert D7 residues, these values decrease significantly, ranging from 2.55 ± 0.38 Å (ISgKAA) to 2.94 ± 0.50 Å (IagKAA). These results indicate that *Pf*DHPS structures remain stable over time if we exclude the flexible insert D7.

#### 2.2.1. Insert D7 Instability

**Figure 3:**
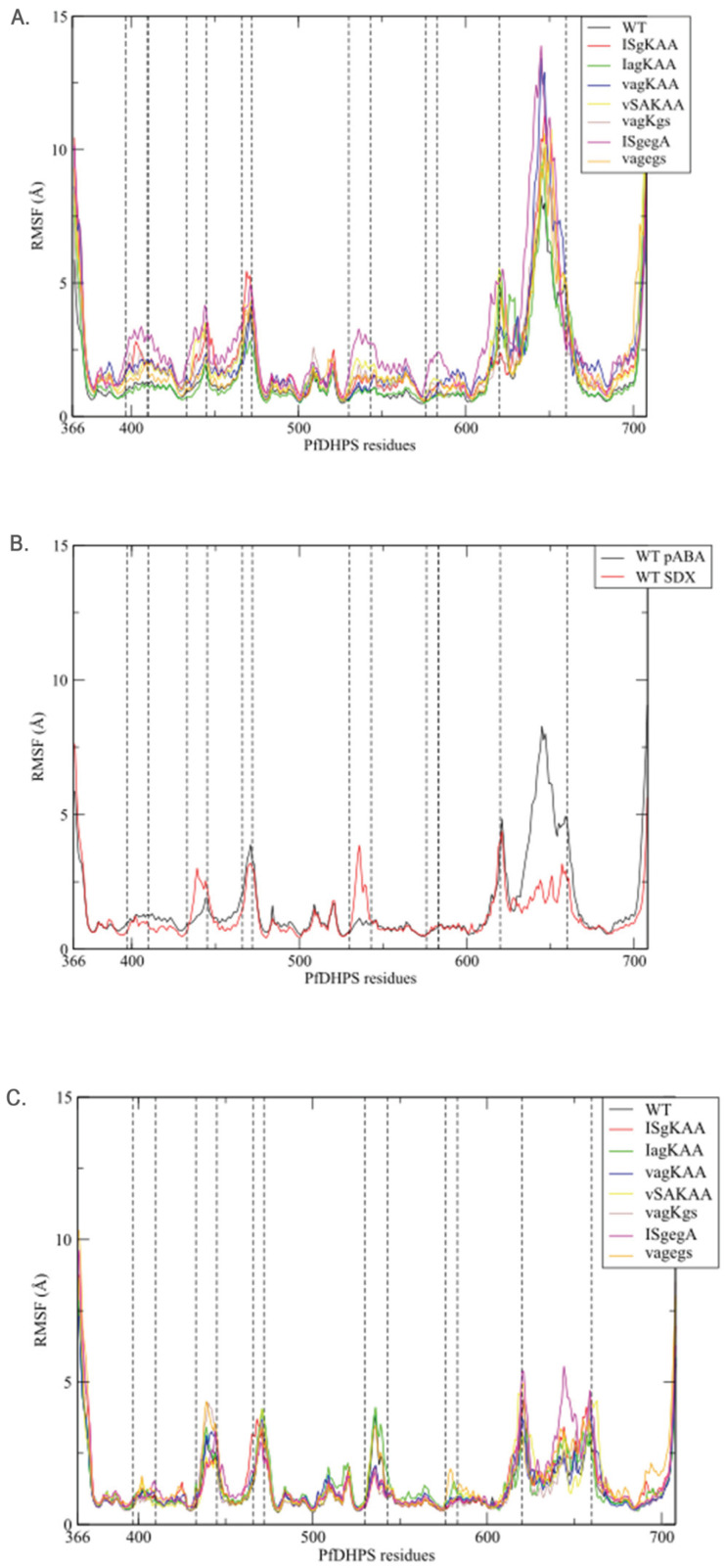
Root mean square fluctuation (RMSF) (**A**) of the eight systems bound to natural ligands (*p*-aminobenzoic acid (*p*ABA) and 6-hydroxymethyl-7,8-dihydropterin pyrophosphate) over 200 ns of molecular dynamics simulations, (**B**) of the wild-type (WT) *Pf*DHPS with natural ligands (black) and with sulfadoxine (red) during 200 ns of molecular dynamics simulations, and (**C**) of the eight systems with sulfadoxine (SDX) and 6-hydroxymethyl-7,8-dihydropterin pyrophosphate during 200 ns of molecular dynamics simulations.

The conformational instability of insert D7 (residues 620 to 660) is clearly highlighted by the RMSF analysis (Figure 3). Indeed, among the eight systems bound to the natural ligands DHPPP and *p*ABA, this region consistently exhibits the highest RMSF values. Notably, the WT system displays the lowest flexibility for this sequence (maximum at 8.28 Å), whereas the vagKAA and ISgegA systems show the greatest fluctuations, with maxima of 13.43 Å and 13.90 Å, respectively.

#### 2.2.2. Loop Flexibility

Distinct differences in loop conformational dynamics are observed across the simulated systems. Specifically, loops D1 (residues 397–410), D2 (433–444), D5 (530–547) and D6 (576–583), and the insert N3 (466–472) exhibit varying degrees of flexibility throughout the simulations. The highest fluctuations are observed in the ISgegA system in these regions, with RMSF maxima of 3.36 Å for loop D1, 4.17 Å for D2, 3.28 Å for D5 and 2.40 Å for D6. In contrast, the WT and IagKAA enzymes display comparatively reduced loop flexibility across these regions. In the following section, we explore how the dynamic behavior of these loops relates to the interaction network within the active site.

### 2.3. Conformational Analysis of PfDHPS Active Site in Complex with pABA

For each system, we conducted a detailed analysis of the structural arrangement and stability of the active site. Intermolecular interactions were characterized using IGMPlot (version 3.03) [11], using the cluster representative structure as a reference. To validate the relevance of the identified interactions over time, a native contact analysis was also performed throughout the trajectories, confirming the persistence of key residue-ligand contacts throughout the simulations.

#### 2.3.1. *p*ABA Binding Pocket

**Figure 4:**
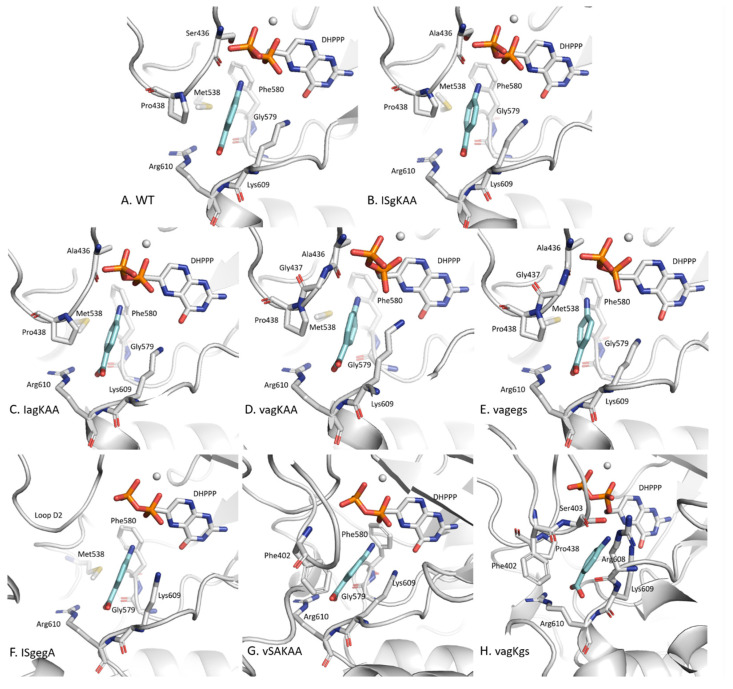
Residues involved in the *p*ABA pocket for *Pf*DHPS variants: (**A**) WT, (**B**) ISgKAA, (**C**) IagKAA, (**D**) vagKAA, (**E**) vagegs, (**F**) ISgegA, (**G**) vSAKAA and (**H**) vagKgs. *p*ABA is shown in blue. The seven residues constituting the *p*ABA pocket are conserved across systems (**A**) to (**E**) and are located on the following loops: Ser436 (or Ala436) and Pro438 (loop D2), Met538 (loop D5), Gly579 and Phe580 on loop D6, Lys609 and Arg610 on loop D7.

IGMPlot data show that seven key residues from the WT system are interacting with *p*ABA (Table S2). All of these residues are located on loops D2 (Ser436 and Pro438), D5 (Met538), D6 (Gly579 and Phe580), and D7 (Lys609 and Arg610). These residues are all found in 4 other systems: ISgKAA, IagKAA, vagKAA and vagegs (Figure 4), forming an identical binding pocket. Native contact analysis also indicates interactions with the residue Ala437, located on loop D2, except for ISgKAA, which is not involved in ISgKAA/*p*ABA interaction. Among these residues, 436 and 437 are known to be point mutations, and residues 538, 579–580 and 609–610 are close to point mutations 540, 581 and 613, respectively.

In the ISgegA system, the *p*ABA binding pocket differs significantly and is characterized by the absence of D2 loop involvement and a reduced number of interactions with the enzyme (Figure 4). Only five residues interact with *p*ABA: Met538 (loop D5), Gly579 and Phe580 (loop D6), and Lys609 and Arg610 (loop D7). This altered interaction network likely contributes to the elevated D2 loop flexibility in the RMSF analysis.

Unlike the other systems, loop D1 contributes to the *p*ABA binding pocket in both the vSAKAA and vagKgs systems. In the vSAKAA system, the main interacting residues are Phe402 (loop D1), Gly579 and Phe580 (loop D6), and Lys609 and Arg610 (loop D7). Native contact analysis further reveals additional interactions with Ser436 (loop D2) and Met538 (loop D5), suggesting a more extensive and stabilizing interaction network in this mutant.

#### 2.3.2. The Distinctive Case of the vagKgs System

Among all systems studied, the vagKgs mutant exhibits the most pronounced differences in the composition of the *p*ABA pocket. Notably, we identify key interacting residues including Phe402 and Ser403 (loop D1), Pro438 (loop D2), and Arg608, Lys609, and Arg610 (loop D7). Native contact analysis also reveals interactions with Ser437 (loop D2), as well as Gly579 and Phe580 (loop D6). While residues Gly579 and Phe580 (loop D6), along with Lys609 and Arg610 (loop D7) are consistently involved in the *p*ABA binding pocket across all systems, Met538 (D5 loop) which participates in the binding with *p*ABA pocket in every system, except vagKgs. These variations suggest a distinct binding mode in this mutant, potentially impacting ligand affinity.

#### 2.3.3. Binding Energies

The above results are consistent with the binding free energy values found for *p*ABA and calculated using the MM/GBSA method (Table 2). The ISgegA system, which involves only five interacting residues, is associated with the weakest binding affinity, with a binding free energy value of −16.51 kcal/mol. In contrast, the vagKgs system, which includes four additional residues, shows the strongest binding energy (−23.60 kcal/mol). The five systems sharing a nearly identical *p*ABA binding pockets (WT, ISgKAA, IagKAA, vagKAA and vagegs) display similar binding free energy values (−19.72 kcal/mol for WT). The ISgKAA system shows the least favorable binding energy (−18.42 kcal/mol), which may be attributed to the absence of residue 437 in the interaction network. Among the systems, all four mutant systems carrying mutations on sites 436 and 437 exhibit increased affinity for *p*ABA.

To further investigate the stability of *p*ABA within the active site, we also monitored the evolution of its position relative to that of DHPPP. Given that *Pf*DHPS catalyzes the condensation of *p*ABA and DHPPP via a covalent bond between the C9 atom of DHPPP and the nitrogen atom of *p*ABA (Figure 1), this interatomic distance was measured across each system over the 200 ns of molecular dynamics simulations and is reported in Table 3. The shortest distances (ranging from 3.43 Å to 3.60 Å) are observed in the four systems carrying the S436A and A437G mutations, coinciding with those previously identified as having an increased affinity for *p*ABA. Among these, the three shortest distances are found in systems that also carry the I431V mutation. These results suggest that specific combinations of mutations, particularly S436A, A437G, and I431V, may have a more favorable positioning of *p*ABA relative to DHPPP.

#### 2.3.4. DHPPP Binding Pocket

**Figure 5:**
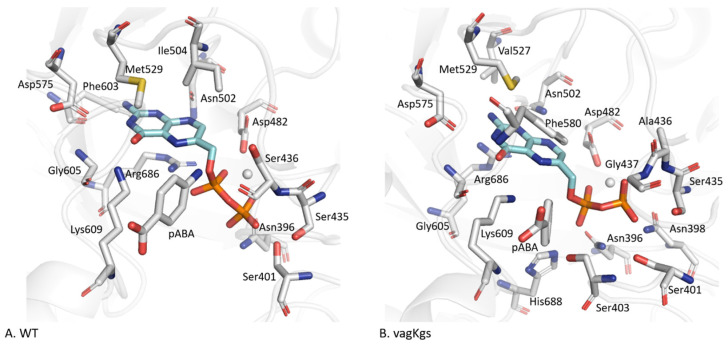
Residues involved in the interaction with DHPPP in (**A**) the wild-type (WT) system and (**B**) the vagKgs variant with natural ligands. DHPPP is shown in blue.

Unlike the residues forming the *p*ABA binding site, those interacting with DHPPP are mainly located on the β-sheet core of the protein (Table S3). In the WT system (Figure 5A), IGMPlot identifies thirteen key residues involved in DHPPP binding: Asn396 (β-sheet β1), Ser401 (loop D1), Ser435 and Ser436 (D2), Asp482 (β3), Asn502 (β4), Ile504 (D4), Met529 (β5), Asp575 (β6), Phe603 (β7), Gly605 and Lys609 (D7) and Arg686 (β8). Native contact analysis further reveals interactions with additional residues, including Glu434, Ala437 (loop D2), Val527 (β-sheet B5) and Phe580 (loopD6). Overall, seven of the eight β-sheets contribute to the DHPPP interaction networks, underlying the structural integration of the binding pocket. Twelve residues are consistently involved in DHPPP binding across all eight systems: Asn396 (β1), Ser435, Ser/Ala436, Ala/Gly437 (D2), Asp482 (β3), Asn502 (β4), Val527 and Met529 (β5), Asp575 (β6), Gly605 and Lys609 (D7) and Arg686 (β8). This interaction network forms an extensive and stable binding pocket, effectively anchoring DHPPP in the catalytic site. Among these, Lys609 (loop D7) appears to be a key residue as it interacts with both *p*ABA and DHPPP in all systems, suggesting its central role in ligand coordination. Conversely, residues Asn398 and Ser403 (loop D1), and His688 (loop D8) are unique to the vagKgs system, suggesting a variant-specific arrangement of the binding environment (Figure 5B).

Finally, we note that residues of inserts N3 and D7, which exhibit significant conformational flexibility according to the RMSF, do not contribute to the binding of the two natural ligands (DHPPP and *p*ABA), further confirming their peripheral role in substrate recognition.

### 2.4. Structural Reorganization of the Active Site in the Case of vagKgs Mutant

**Figure 6:**
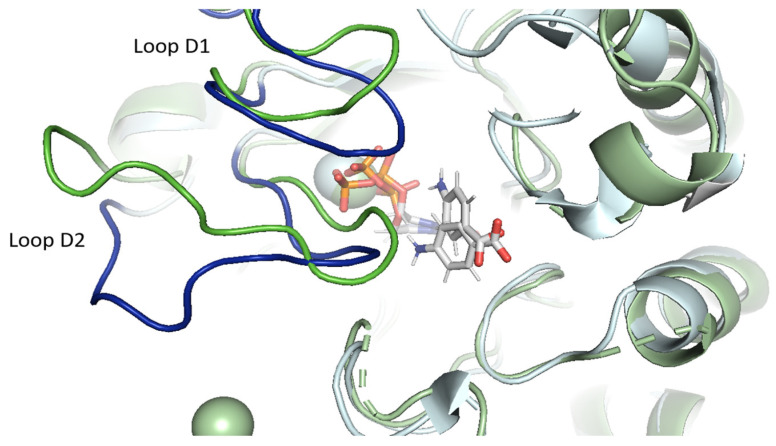
Conformation of D1 and D2 loops after clustering for *Pf*DHPS wild-type (green) and vagKgs mutant (blue). Structural alignment was performed in PyMOL using the *align* command, which performs sequence alignment and superimposes the α-carbon atoms.

In the vagKgs mutant, a substantial reorganization of the active site is observed, compared to the WT enzyme (Figure 6). This structural rearrangement is primarily associated with conformational changes in the D1 and D2 loops. Indeed, four additional residues, Asn398, Ser403, Asp482 and His 688, are positioned in the vicinity of DHPPP and form stabilizing hydrogen bonds (Figure 5B). We note that the conformations adopted by the D1 and D2 loops in the vagKgs system differ not only from the WT, but also from all the other systems studied, highlighting a unique architecture in this mutant. This reorganization may have functional implications for ligand binding and catalysis.

### 2.5. Comparison in Presence of Sulfadoxine

#### 2.5.1. Sulfadoxine Instability

The same study was carried out by replacing *p*ABA with SDX. However, the simulation times in the presence of SDX were significantly shortened in all systems, due to early dissociation of the inhibitor from the active site. This rapid unbinding was consistently observed, indicating reduced binding stability compared to the natural ligand. However, in the vagKgs system, SDX exhibits a distinct behavior: after leaving its original binding pocket, the inhibitor migrated to a nearby site within the active site and remained stably bound over the rest of the simulation, unlike other systems (Figure S4). In the vagKgs system, the removal of SDX from its original binding site does not appear to interfere with the anchoring of *p*ABA in the active site. However, the presence of SDX induces a conformational change in the D2 loop, which adopts an open conformation. The residues interacting with SDX in this alternate position do not differ significantly (as discussed in the following paragraph) and include Phe402 (loop D1), Pro438 and Phe439 (D2), Pro535 (D5), and Val621 and Ile623 (insert D7). The displacement of SDX in the enzyme was monitored by measuring the distance between the atoms C9 of DHPPP and the nitrogen atom of SDX throughout the trajectory (Figure S5).

#### 2.5.2. Conformational Variability of Sulfadoxine

Interestingly, we also observe that SDX adopts different conformations throughout the simulations. Analysis of the dihedral angles between the rings of SDX reveals a correlation between its position within the enzyme and its conformational stability. Figures S5 and S6 depict the variations of the C18-S21-N22-C25 dihedral angle, showing that SDX transits among three distinct conformational states: an initial state around −20°, followed by fluctuations between this state and two alternative states around +120° and −130° when SDX leaves the active site. Notably, the vagKgs variant shows distinct behavior, with the dihedral angle predominantly stabilized near −20°, rarely sampling the +120° conformation. Similarly, the ISgegA system does not explore this +120° state, but predominantly adopts the −130° conformation. In order to explore SDX conformational landscape, we performed a scan of the C18-S21-N22-C25 dihedral angle using the semi-empirical tight-binding method GFN2-xTB [12]. We characterized three local minima very similar to the three states identified in the MD simulations (Figure S7). Thus, the starting conformation does not correspond to the most stable minimum found with GFN2-xTB. This suggests that the enzyme environment imposes conformational constraints that favor suboptimal but structurally accommodated SDX conformers. Such a deviation from the lowest-energy geometry may contribute to reduced binding affinity and premature dissociation observed across systems.

#### 2.5.3. Enzyme Stability

As observed with the natural ligands, the total energy of all eight systems complexed with SDX remained stable throughout the 200 ns production phase, ensuring the overall stability of the simulations (Figure S8). The RMSD values, calculated across all residues (Figure S9), with mean values ranging from 2.68 ± 0.36 Å for the vagKAA system to 3.72 ± 0.60 Å for ISgegA (Table 4), indicate overall structural stability.

A comparison of RMSF profiles of WT *Pf*DHPS bound either to the natural ligands (DHPPP and *p*ABA) or to the inhibitor (DHPPP and SDX) reveals three notable differences (Figure 3B). In the presence of SDX, the D2 and D5 loops exhibit increased flexibility, while the D7 insert, previously more mobile with *p*ABA, is associated with a significant reduction in flexibility. This decreased mobility may be attributed to specific stabilizing interactions between SDX and residues within the insert D7 region, as discussed below. Importantly, this trend is consistent across all eight systems bound to SDX: they exhibit similar RMSF profiles, consistently showing decreased mobility in the D7 insert compared to *p*ABA bound systems (Figure 3C). These observations suggest that, although SDX is prone to dissociation, its transient presence may still influence local structural dynamics, not only in the active site but also in the more distant insert D7 region.

### 2.6. Conformational Analysis of PfDHPS Active Site in Complex with Sulfadoxine

#### 2.6.1. WT *Pf*DHPS

For the WT system, we conducted a detailed analysis of the active site structure and stability in the presence of SDX, focusing on the first 130 ns of the molecular dynamics trajectory. Intermolecular interactions were analyzed using IGMPlot, based on the representative structure from the main cluster as a reference. We also performed a native contact analysis throughout the trajectories to confirm the persistence of key interactions identified with IGMPlot.

**Figure 7:**
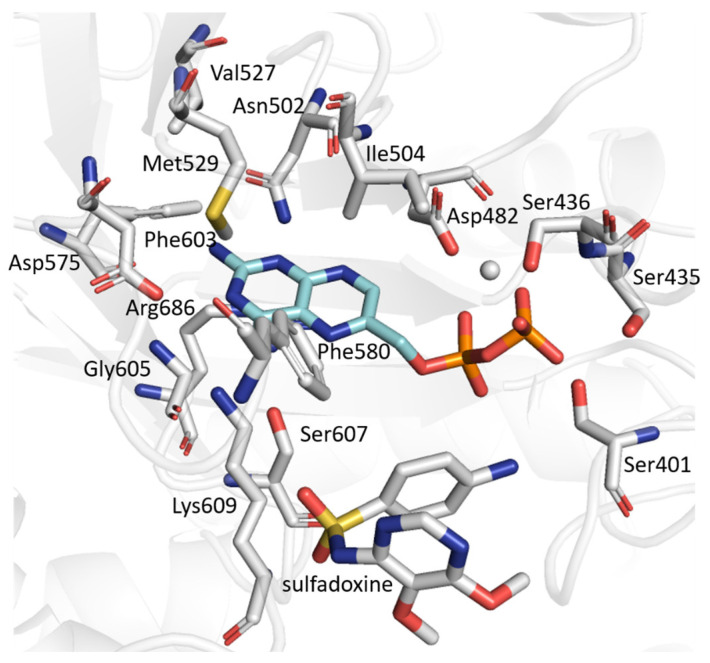
Residues involved in the interaction with DHPPP in the wild-type *Pf*DHPS bound to sulfadoxine.

The residues involved in DHPPP binding are mostly conserved whether the *Pf*DHPS is complexed with the natural ligand *p*ABA or the inhibitor SDX (Table S4, Figure 7). One notable difference is the presence of Glu434, which is only involved in the *p*ABA-bound system. Conversely, residues Ile394, Phe402 and Gly605 are only involved in the presence of SDX. We conclude that the interaction with the natural ligand (*p*ABA) or the inhibitor (SDX) does not alter the DHPPP/DHPS interaction network.

**Figure 8:**
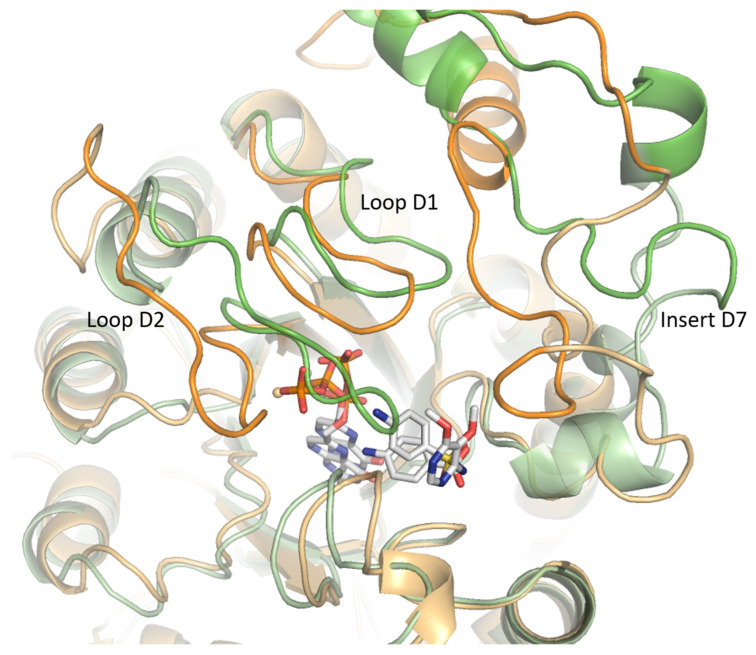
Conformations of the D1 and D2 loops, and the D7 insert, after clustering for *Pf*DHPS WT bound. to *p*ABA (green) and sulfadoxine (orange). Structural alignment was performed in PyMOL (version 2.5.2) using the *align* command, which combines sequence alignment with superposition of α-carbon atoms.

Native contact analysis reveals that six key residues in the WT enzyme maintain stable interactions with SDX for more than 50% of the simulation time (Table S5). These residues are located on loops D1 (Phe402), D5 (Pro535 and Met538), D6 (Phe580), and D7 (Lys609 and Arg610). IGMPlot analysis of the representative cluster frame further identifies transient interactions involving the residue Gly579 (loop D6), as well as Val622 and Asn624, from the N-terminal region of the D7 insert. Residues Val622 and Asn624 interact with SDX for 27% and 23% of the simulation time, respectively. Due to the structural similarity of the aminobenzene moiety in *p*ABA and SDX, the interaction profiles of both ligands within the WT systems are largely conserved (Table S5). The main difference lies in the loss of interactions involving D2 loop residues in the presence of SDX (Figure 8). Loops D1 and D2 display marked conformational flexibility, adopting distinct conformations depending on the bound ligand. Specifically, the D2 loop closes in the presence of the natural ligands (DHPPP and *p*ABA) but shifts to an open conformation upon SDX binding. This rearrangement likely underlies the reduced binding stability and premature dissociation of the inhibitor.

#### 2.6.2. *Pf*DHPS Mutants

We conducted a detailed analysis of SDX interactions in mutated *Pf*DHPS where SDX remained in its original binding site for over 50 ns. The systems studied include ISgKAA, IagKAA, vagKAA and vagKgs. IGMPlot data and native contact analysis reveals that key residues—Phe580 (in loop D6), Lys609 and Arg610 (both in loop D7)—consistently interact with SDX across all four systems (Figure S10 and Table S6). The IagKAA system shows the most frequent interactions. Unlike the WT system, loop D2 also contributes to the SDX binding pocket in all the four systems, with the involvement of residues 436, 437 and/or 438.

Binding free energies for SDX were calculated only for mutated *Pf*DHPS systems in which SDX remained in the binding pocket for at least 50 ns (which excludes vSAKAA, ISgegA and vagegs). Although this sampling window is shorter than the one used for *p*ABA, extending the analysis beyond the dissociation event would not be meaningful. In this context, the 50 ns window provides the most consistent and informative estimate of the bound state. However, it should also be emphasized that MM/GBSA results are highly dependent on the quality of conformational sampling, and longer trajectories would provide more reliable estimates of binding free energy. A 50 ns sampling window is short and does not allow for an accurate estimation of equilibrium properties. The higher standard deviations observed compared to the *p*ABA systems reflect this more limited sampling and do not allow us to conclude about a gain or loss of stability (Table 5).

## 3. Discussion

The crystal structure of *Pf*DHPS was only resolved recently and still contains several unresolved regions, particularly within the parasite-specific inserts [3]. Earlier simulation studies were based on homology models that excluded two [8] or one [9] of these inserts. Residues 612–670, absent from the crystal structure 6JWQ, were extracted from a homology model. However, the confidence level for this region was low, as indicated by a QMEAN score of 0.567 [13]. Molecular dynamics simulations reported by Boateng et al. [14], using PDB ID 6JWX [3], showed RMSF profiles for SDX-bound *Pf*DHPS broadly consistent with our results. However, a key difference emerges in the flexibility of this insert in the absence of inhibitor. In our simulations, this region displays markedly greater mobility in the *p*ABA-bound state, suggesting a ligand-induced conformational response. This observation also supports the hypothesis that insert D7 can be considered structurally independent from the enzyme core, particularly in the monomeric form used in our simulations. Several studies have indicated that parasite-specific inserts are implicated in the function and structural stabilization of *P. falciparum* bifunctional enzymes, including *Pf*HPPK-DHPS [15]. Our findings are consistent with these reports, indicating that while these inserts do not directly interact with the natural substrates (DHPPP or *p*ABA), they may play structural or regulatory roles. A more detailed structural and functional characterization of this insert would be valuable for understanding its contribution to enzyme architecture and drug resistance mechanisms.

Excluding insert D7, RMSF analyses reveal no significant differences in overall *Pf*DHPS stability across mutant systems. As expected, the introduced mutations do not compromise global enzyme integrity, which is a prerequisite for catalytic function, and by extension, for parasite viability. This also applies to the vagegs system, which, although not yet reported in field studies, cannot be excluded as a potential future variant. This study does not preclude the possible emergence of this allele under selective pressure. However, while the enzyme remains globally stable, local rearrangements within the active site can be substantial, as exemplified by the vagKgs mutant.

A conformational analysis of the *Pf*DHPS active site across WT and mutant systems bound to *p*ABA reveal both conserved and divergent features influencing ligand stability. In the WT and four variants (ISgKAA, IagKAA, vagKAA, vagegs), the *p*ABA binding pocket remains largely intact, relying on key residues from loops D2, D5, D6, and D7, highlighting the critical role of D2 loop contacts in maintaining ligand binding. The only exception is ISgKAA, the only variant where residue 437 is mutated while 436 remains unchanged. The conservation of this binding pocket in multiple mutants indicates a degree of structural resilience in *p*ABA recognition when D2 loop contacts are maintained.

In contrast, the ISgegA system shows a disrupted binding pocket: the D2 loop does not participate in ligand stabilization, and the interaction network is limited to five residues, correlating with the increased D2 loop flexibility and lower *p*ABA binding affinity. These findings underscore the functional importance of the D2 loop in stabilizing substrate binding and suggest that its disruption may be a key mechanism in resistance.

Two other mutants, vSAKAA and vagKgs, show a restructured binding pocket that incorporates loop D1, suggesting a compensatory rearrangement allowing loop D1 to support ligand stabilization when D2 contacts are weakened.

Our results also suggest that specific combinations of mutations, particularly S436A, A437G, and I431V, may enhance the catalytic efficiency of *Pf*DHPS by promoting a more favorable positioning of *p*ABA relative to DHPPP. The reduced distance between the reactive atoms could facilitate covalent bond formation, which is a critical step in the catalytic mechanism. Hybrid Quantum Mechanical/Molecular Mechanical (QM/MM) calculations should be conducted to test this hypothesis by comparing the energy barriers.

Altogether, these observations highlight the structural plasticity of the *p*ABA binding pocket and its capacity to adapt to mutations through alternative stabilizing interactions. The D2 loop emerges as a central determinant of ligand affinity, with its involvement—or lack of involvement—strongly influencing binding strength. Additionally, the integration of loop D1 into the binding pocket in specific mutants underscores the enzyme conformational adaptability, with implications for resistance evolution and inhibitor design.

These results can be related to the dynamic behavior of loops D1 and D2, which adopt distinct conformational states depending on the bound ligand. Specifically, while the D2 loop tends to adopt a closed conformation around natural substrates (DHPPP and *p*ABA), it shifts toward an open conformation in the presence of SDX. This observation is consistent with the previous structural study reported by Chitnumsub et al. [3].

In this case, we have also highlighted the marked instability of SDX across all systems, particularly in mutants. Boateng et al. [14] previously reported that, in ISAKgA, ISAKAs and ISgKgs mutants, SDX tends to dissociate from the active site or migrate toward an adjacent binding pocket, while remaining stable in the WT system over 150 ns. In contrast, our extended 200 ns simulations reveal that even in the WT system, SDX dissociates after 130 ns, suggesting that mutations not only reduce SDX binding stability but may also compromise its inhibitory efficacy, potentially contributing to resistance mechanism.

We conclude that the ligand-induced D2 loop opening likely contributes to the premature unbinding of SDX observed in our simulations. The native contact analysis confirms this trend: while *p*ABA relies on an extensive network as described above, SDX binding is more localized, and often restricted to a subset of residues on D6 and D7. Moreover, the dihedral angle analysis of SDX reveals conformational variability that may further hinder its stable accommodation in the binding pocket. From a drug design perspective, this highlights a key limitation of SDX: its structural flexibility and sensitivity to the active site environment may limit its effectiveness against *Pf*DHPS variants. Future antifolate inhibitors could benefit from conformational preorganization, locking key torsional angles into bioactive geometries, to improve affinity and specificity, even in the presence of resistance-associated mutations.

## 4. Materials and Methods

### 4.1. Molecular Structures

The initial structure of the WT *Pf*DHPS protein (residues from Ile 366 to Asp 708) was extracted from the Protein Data Bank (PDB) entry 6JWQ [3]. To complete the missing parts, the positions of residues 397–410, 436–445 and 612–670 were extracted from the PDB entries 5Z79 (*Plasmodium vivax*) [16], 6JWR (*Plasmodium falciparum*) and from a homology model, respectively [13]. The PDB entry 5Z79 from *P. vivax* HPPK-DHPS was used for sequence conservation, but residues 407 (leucine 407 with isoleucine) and 410 (aspartic acid 410 with glutamine) were modified in accordance with the *Pf*DHPS WT sequence (Figure S11).

The obtained structure was compared with the AlphaFold2 prediction (version 2.2.0) [10]. All structure alignments were performed using PyMOL (version 2.5.2) [17] and the align command, which conducts both sequence alignment and structural superposition of the α-carbon atoms.

### 4.2. System Preparation

A total of seven mutants was generated from the WT *Pf*DHPS structure obtained after MD production, using the AMBER package (version 16) [18], incorporating the following substitutions: isoleucine 431 to valine (Ile431Val), serine 436 to alanine (Ser436Ala), alanine 437 to glycine (Ala437Gly), Lysine 540 to glutamine (Lys540Glu), alanine 581 to glycine (Ala581Gly), and alanine 613 to serine (Ala613Ser) as described in the Supplementary Table S1. Five alleles were selected on the basis of epidemiological data (ISgKAA, IagKAA, vagKAA, vagKgs, ISgegA). The latter two alleles were not found in epidemiological studies: the isolated Ile431Val mutation (vSAKAA) and a combination of all six mutations (vagegs). To date, the combination of these six mutations has not been reported in epidemiological studies. Investigating these alleles will provide insights into why they have not been observed in the field and whether they might emerge in the future.

DHPPP and *p*ABA structures were extracted from the PDB entry 6JWR. SDX structure was obtained from PDB entry 6JWX. A Mg^2+^ cofactor coordinates the diphosphate group within DHPPP.

### 4.3. Molecular Dynamics Simulations

All MD simulations were performed with the AMBER package, using the ff14SB force-field [19].

Ligand parameters were generated using the General Amber Force Field (GAFF) [20] and prepared with the Antechamber programs [21]. The Leap module was used to prepare the *Pf*DHPS/ligand complexes.

All the systems were solvated in a cubic box with a minimal distance of 8.0 Å between the protein and the box edge, using the TIP3P water model [22]. The total charge of the system was neutralized with Cl^−^ ions.

For each complex, an energy minimization was first performed with a 20 kcal.mol^−1^.A^2^ constraint applied to the solute. A second minimization without restraint was then performed on the whole system. Subsequently, the entire system was heated from 100 to 300 K during 500 ps using a 2 fs time step and a Langevin thermostat with a collision frequency of 2 ps^−1^ at constant volume. The restraints initially applied on the complex were slowly released during the equilibration process, run for 500 ps at constant pressure to allow the system density to be stabilized, with 1 atm target pressure using the Berendsen barostat with a pressure relaxation time of 2 ps. Finally, the production setup was implemented in the NVT ensemble (i.e., the number of particles N, the volume V, and the temperature T of the system are kept constant). Long-range electrostatic interactions were treated using the particle–mesh Ewald method with an 8.0 Å cutoff, while covalent bonds involving hydrogen atoms were constrained using the SHAKE algorithm with a 2 fs integration time step.

MD simulations were performed for a total of 200 ns to ensure sufficient sampling of the protein-ligand complex, allowing for the accurate exploration of conformational changes, dynamic interactions and the evaluation of ligand stability within the binding site. Structural coordinates were written after every 2 ps, resulting in a total of 100,000 frames. Analyses of root mean square deviation (RMSD), root mean square fluctuations (RMSF), distances and hydrogen bonds were performed using the AMBER module CPPTRAJ [23]. The Visual Molecular Dynamics (VMD) software (version 1.9.4a53) was used to visualize the trajectories [24].

Mutated systems were prepared after 10 ns of WT system production and extraction of the cluster most representative structure.

### 4.4. MD Trajectory Analysis

The binding free energies were calculated using molecular mechanics with generalized Born surface area (MM/GBSA) [25]. For each system, 98,000 snapshots of the last 198 ns were used on the MD trajectory.

MD simulations analysis (root mean square deviation (RMSD) and root mean square fluctuation (RMSF)) were performed using the CPPTRAJ utility of AMBER [23].

The clustering was performed on each trajectory to extract significant structures as starting points for the active site characterization using the density-based spatial clustering of applications with noise method (DBSCAN), with 25 as the minimum number of points required to form a cluster and a distance threshold of 0.9 Å between points to form a cluster [26].

To confirm intramolecular interactions between *Pf*DHPS and ligands during MD simulations, the nativecontacts function from the CPPTRAJ utility of AMBER was used, with a maximum distance of 4.0 Å [23]. Only interactions present in more than 50% of frames were retained.

### 4.5. IGMPlot

IGMPlot with a grid increment of 0.05 Å in each direction was used to assess intramolecular interactions between *Pf*DHPS and ligands [11].

## 5. Conclusions

This study provides a comprehensive view of *Pf*DHPS dynamics and its interactions with both natural ligands and SDX, across WT and resistance-associated mutants. Using molecular dynamics simulations, we show that while the global structural stability of *Pf*DHPS is maintained despite mutations, local conformational changes, particularly involving loop D2, deeply affect ligand binding.

The D2 loop emerges as a central determinant of ligand affinity. Among all the systems that exhibit their own specificities, we highlighted the distinctive case of the vagKgs mutant, in which the compensatory recruitment of loop D1 contributes to enhancing the natural ligand stabilization. In contrast, SDX consistently fails to engage D2 residues and displays increased conformational variability, leading to early unbinding events and reduced interaction networks, especially in the mutant systems.

Our findings reinforce the critical role of structural plasticity in resistance evolution and highlight specific regions as strategic targets for inhibitor design. Mimicking the natural binding mode of *p*ABA, especially its interaction with loop D2, or exploiting stabilizing contacts within more conserved structural regions may offer a path toward designing new antifolates that remain effective against resistant strains.

Altogether, this study not only explores the molecular basis of SDX resistance in *Pf*DHPS but also emphasizes the delicate interplay between active site accommodation upon mutations, ligand selectivity, and catalytic efficiency. In this regard, this study paves the way to further investigation into the catalytic activity of *Pf*DHPS. This study is currently under investigation within our team in order to assess how mutations impact enzymatic activity, using hybrid Quantum Mechanical/Molecular Mechanical calculations.

## Figures and Tables

**Table 1:** Root mean square deviation (RMSD) of the eight *Pf*DHPS systems bound to their natural ligands over 200 ns of molecular dynamics simulations, calculated with and without inclusion of the insert-D7 residues.

	With Insert D7	Without Insert D7
	Average ± Std. Dev. (Å)	Average ± Std. Dev. (Å)
WT	6.20 ± 0.69	2.73 ± 0.23
ISgKAA	4.85 ± 1.04	2.55 ± 0.38
IagKAA	4.91 ± 0.93	2.94 ± 0.50
vSAKAA	4.08 ± 0.73	2.87 ± 0.51
vagKAA	4.94 ± 1.63	2.69 ± 0.41
vagKgs	3.58 ± 0.63	2.59 ± 0.33
ISgegA	4.56 ± 1.85	2.80 ± 0.51
vagegs	4.28 ± 1.16	2.92 ± 0.57

**Table 2:** Binding free energies of *p*ABA calculated using Molecular Mechanics/Generalized Born Surface Area (MM/GBSA) method. For each system, 98,000 snapshots from the last 198 ns of the molecular dynamics trajectory were used for the calculation.

	Average (kcal/mol)	Std. Dev. (kcal/mol)
WT	−19.72	2.85
ISgKAA	−18.42	3.42
IagKAA	−20.43	2.79
vSAKAA	−18.41	3.96
vagKAA	−19.79	3.86
vagKgs	−23.60	3.69
ISgegA	−16.51	4.89
vagegs	−20.33	2.74

**Table 3:** Distance between the C9 atom of 6-hydroxymethyl-7,8-dihydropterin pyrophosphate and the nitrogen atom of *p*-aminobenzoic acid, measured throughout the 200 ns molecular dynamics simulations for each system.

	Average (Å)	Std. Dev. (Å)
WT	3.64	0.32
ISgKAA	3.83	0.44
IagKAA	3.60	0.30
vSAKAA	3.82	0.77
vagKAA	3.45	0.28
vagKgs	3.43	0.25
ISgegA	3.84	0.47
vagegs	3.45	0.24

**Table 4:** Root mean square deviation (RMSD) of the eight *Pf*DHPS systems in the presence of the inhibitor (sulfadoxine) during 200 ns of molecular dynamics simulations.

	Average ± Std. Dev. (Å)
WT	2.69 ± 0.36
ISgKAA	3.42 ± 0.38
IagKAA	3.18 ± 0.38
vSAKAA	2.96 ± 0.31
vagKAA	2.68 ± 0.36
vagKgs	2.79 ± 0.39
ISgegA	3.72 ± 0.60
vagegs	3.36 ± 0.59

**Table 5:** Binding free energies of SDX calculated using Molecular Mechanics/Generalized Born Surface Area (MM/GBSA) method. For each system, 28,000 snapshots from the first 50 ns of the molecular dynamics trajectory were used for the calculation.

	Average (kcal/mol)	Std. Dev. (kcal/mol)
WT	−19.06	3.56
ISgKAA	−16.67	5.58
IagKAA	−20.24	4.45
vagKAA	−17.94	5.80
vagKgs	−18.92	4.03

## Data Availability

The original contributions presented in this study are included in the article/Supplementary Materials. Further inquiries can be directed to the corresponding author.

## Supplementary Materials

The following supporting information can be downloaded at: https://www.mdpi.com/article/10.3390/molecules30204118/s1, Figure S1: Structure of Wild-Type *Plasmodium falciparum* dihydropteroate synthase; Figure S2: Total energy profiles for the eight *Pf*DHPS systems bound to their natural ligands; Figure S3: RMSD profiles of all *Pf*DHPS residues and of residues excluding the insert D7; Figure S4: Position of Sulfadoxine after unbinding; Figure S5: Distance between the C9 atom of DHPPP and the nitrogen atom of Sulfadoxine; Figure S6: Dihedral angle between atoms C18-S21-N22-C25 of Sulfadoxine; Figure S7: Energy profile for the scan of the C18-S21-N22-C25 dihedral angle in Sulfadoxine; Figure S8: Total energy profiles of the eight *Pf*DHPS systems in the presence of Sulfadoxine; Figure S9: RMSD profiles of all *Pf*DHPS residues across the eight systems in the presence of Sulfadoxine; Figure S10: Residues involved in the interaction with Sulfadoxine for *Pf*DHPS variants; Figure S11: Description of *Pf*DHPS structure building; Table S1: List of the studied systems with the corresponding mutations of interest; Table S2: Residues involved in the *p*ABA pocket; Table S3: Residues involved in the interaction of *Pf*DHPS with DHPPP in systems with natural ligands; Table S4: Residues involved in the interaction of *Pf*DHPS with DHPPP in the WT systems; Table S5: Residues involved in the interaction of *Pf*DHPS with *p*ABA or Sulfadoxine in the WT systems; Table S6: Residues involved in the interaction of *Pf*DHPS with Sulfadoxine.

## Abbreviations

The following abbreviations are used in this manuscript:

DHFR	dihydrofolate reductase
DHP	7,8-dihydropteroate
DHPPP	6-hydroxymethyl-7,8-dihydropterin pyrophosphate
DHPS	dihydropteroate synthase
HPPK	6-hydroxymethyl-7,8-dihydropterin pyrophosphokinase
MD	molecular dynamics
*p*ABA	*p*-aminobenzoic acid
PDB	protein data bank
SP	sulfadoxine–pyrimethamine
SDX	sulfadoxine
