# Supplementary material for: Deciphering the Impact of Mutations on PfDHPS Active Site and Sulfadoxine Binding: Structural Insights from Molecular Dynamics Simulations
Source: Molecules. 2025 Oct 17;30(20):4118. doi: 10.3390/molecules30204118 (PMC12566285; doi:10.3390/molecules30204118)

Supplementary Materials

Deciphering the Impact of Mutations on *Pf*DHPS Active Site and Sulfadoxine Binding: Structural Insights from Molecular Dynamics Simulations

Emilie Guémas [1,2,3,*], Sandie Ménard [2], Nicolas Jeanne [1,2], Georges Landa [1], Antoine Berry [2,3] and Marie Brut [1]

1 Laboratoire d'Analyse et d'Architecture des Systèmes (LAAS)-CNRS, Université de Toulouse, CNRS, 31031 Toulouse, France; jeanne.n@chu-toulouse.fr (N.J.); georges.landa@laas.fr (G.L.); marie.brut@laas.fr (M.B.)
2 Institut Toulousain des Maladies Infectieuses et Inflammatoires (Infinity), Université de Toulouse, CNRS UMR 5051, INSERM UMR 1291, 31024 Toulouse, France; sandie.menard@inserm.fr (S.M.); berry.a@chu-toulouse.fr (A.B.)
3 Service de Parasitologie et Mycologie, Centre Hospitalo-Universitaire (CHU) Toulouse, 31300 Toulouse, France
* Correspondence: guemas.e@chu-toulouse.fr

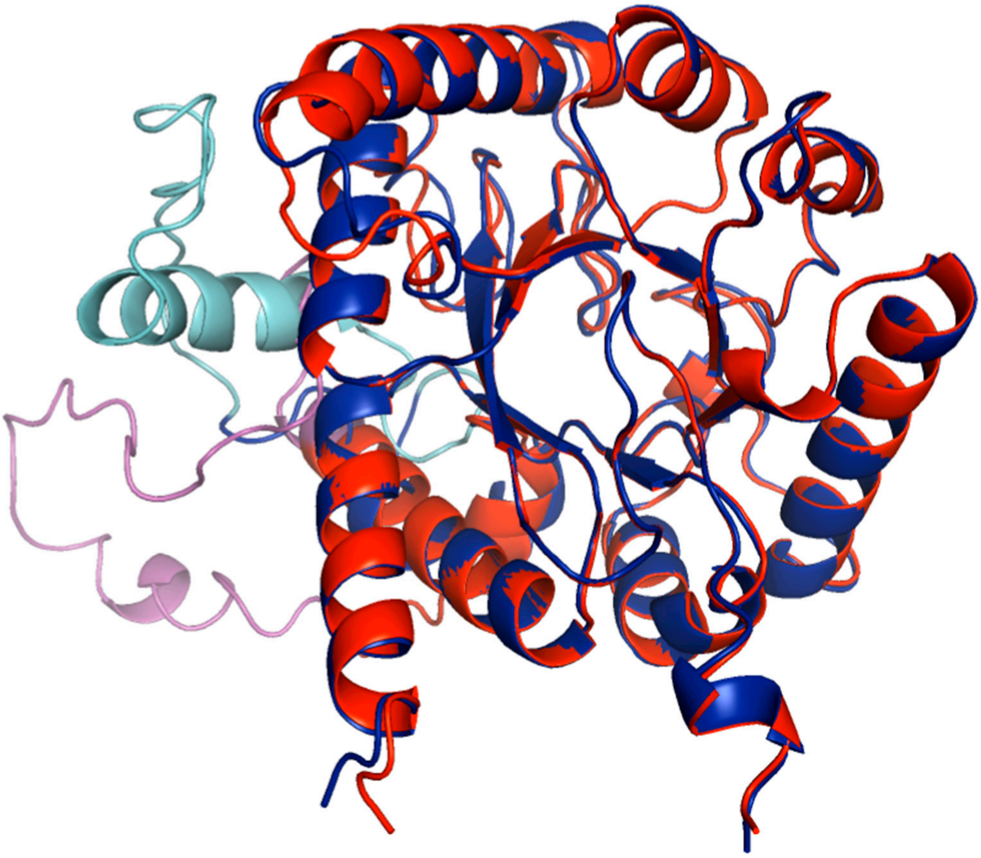

Figure S1. Structure of Wild-Type *Plasmodium falciparum* dihydropteroate synthase. Wild-type *Pf*DHPS structure was obtained by complementing the incomplete regions of PDB entry 6JWQ with structural data from 5Z79 (*P. vivax*), 6JWR (*P. falciparum*) and a homology model of *Pf*DHPS published by Oguike *et al.* (shown in blue). The resulting model was compared to the Alpha Fold2 prediction (shown in red). Structural differences between the two models (highlighted in cyan and pink) are located within the D7 insert region (residues 620 to 660). Structural alignment was performed using PyMOL *align* command, which combines sequence alignment and superposition of α-carbon atoms. The alignment yields a root mean square deviation of 0.260 angstroms over 252 aligned residues

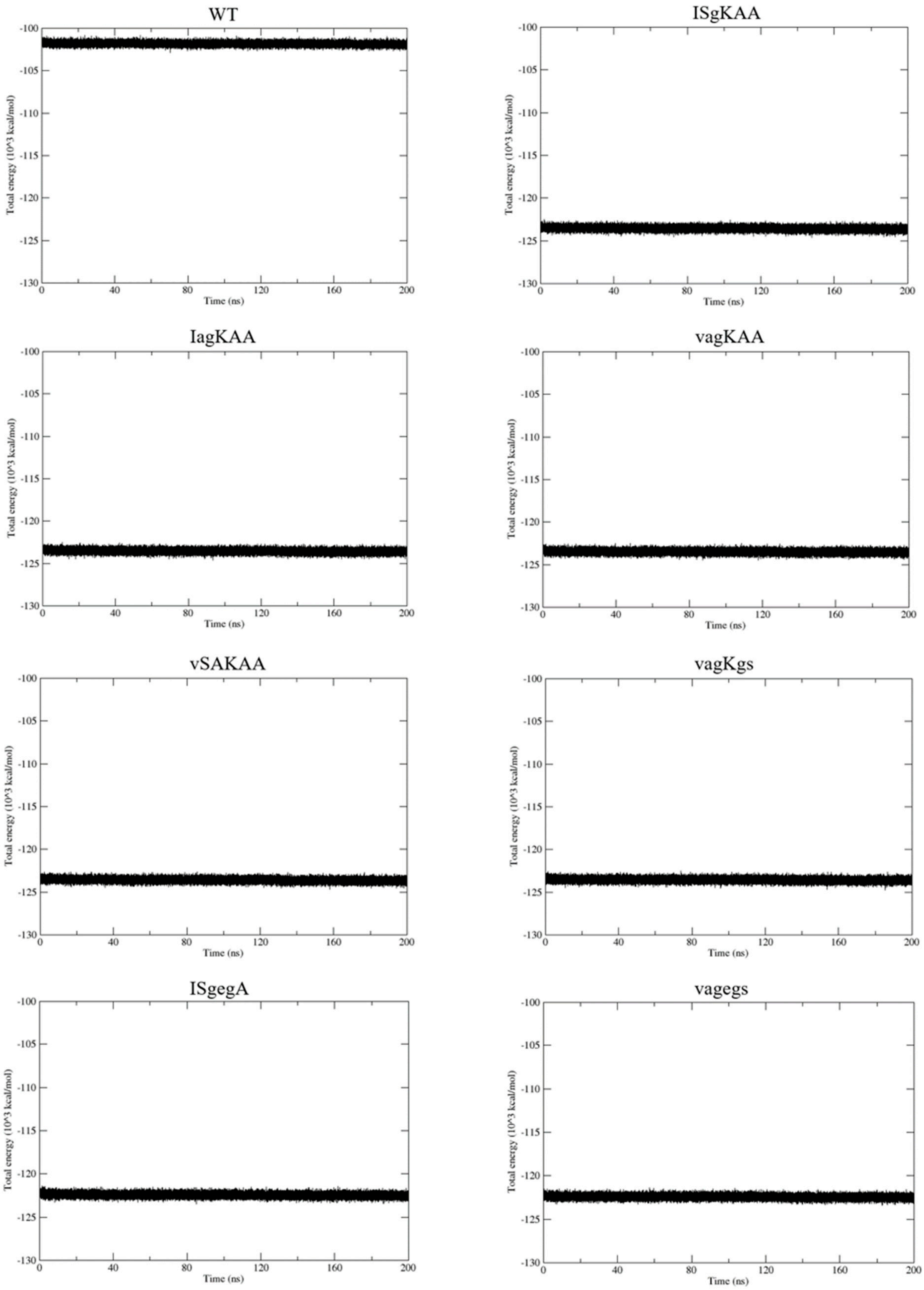

Figure S2. Total energy profiles for the eight *Pf*DHPS systems bound to their natural ligands over 200 ns of molecular dynamics simulations.

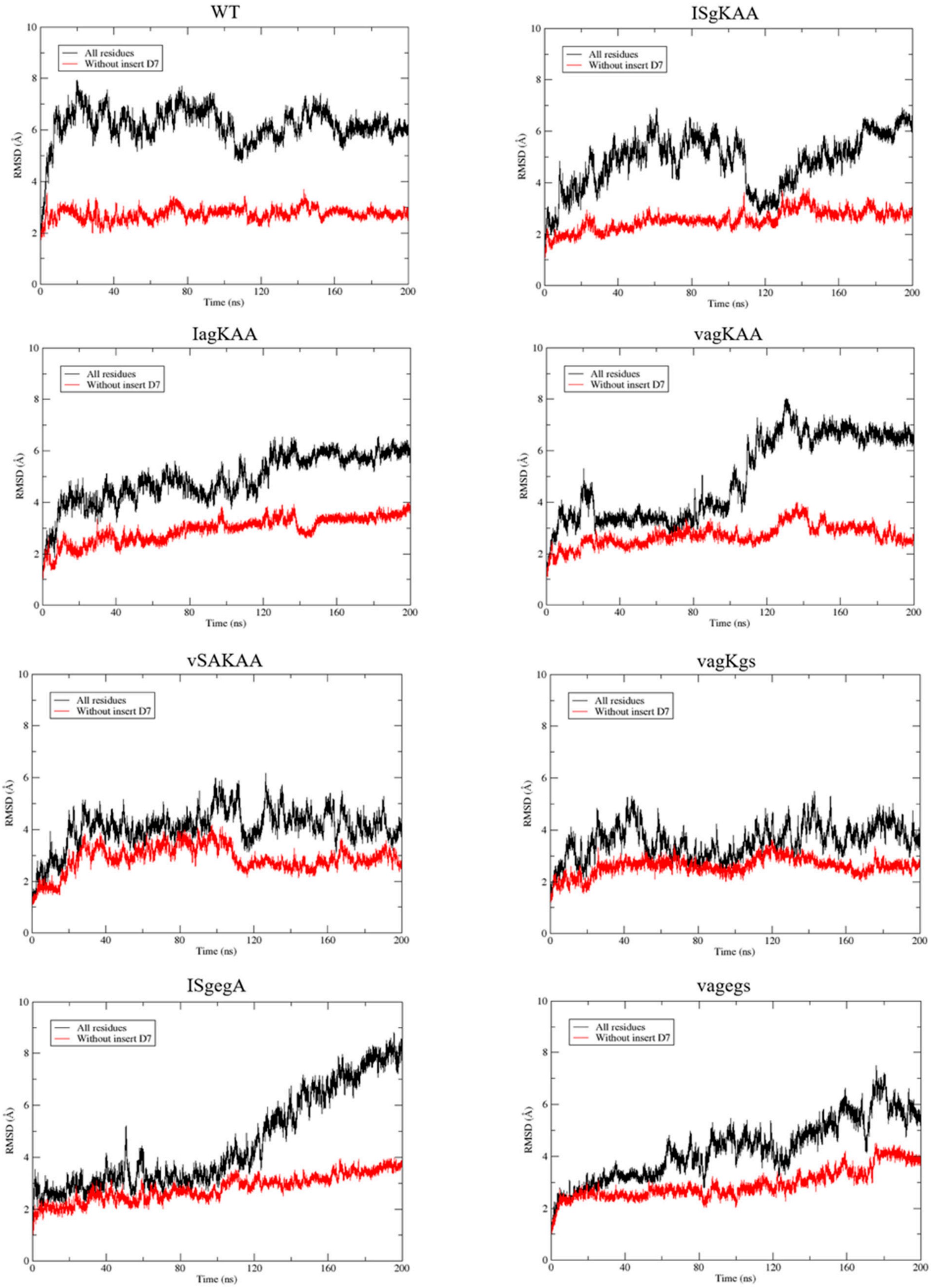

Figure S3. RMSD profiles of all *Pf*DHPS residues (black) and of residues excluding the insert D7 (residues 620-660) (red), across eight systems, each bound to its natural ligand, over 200 ns of molecular dynamics simulations

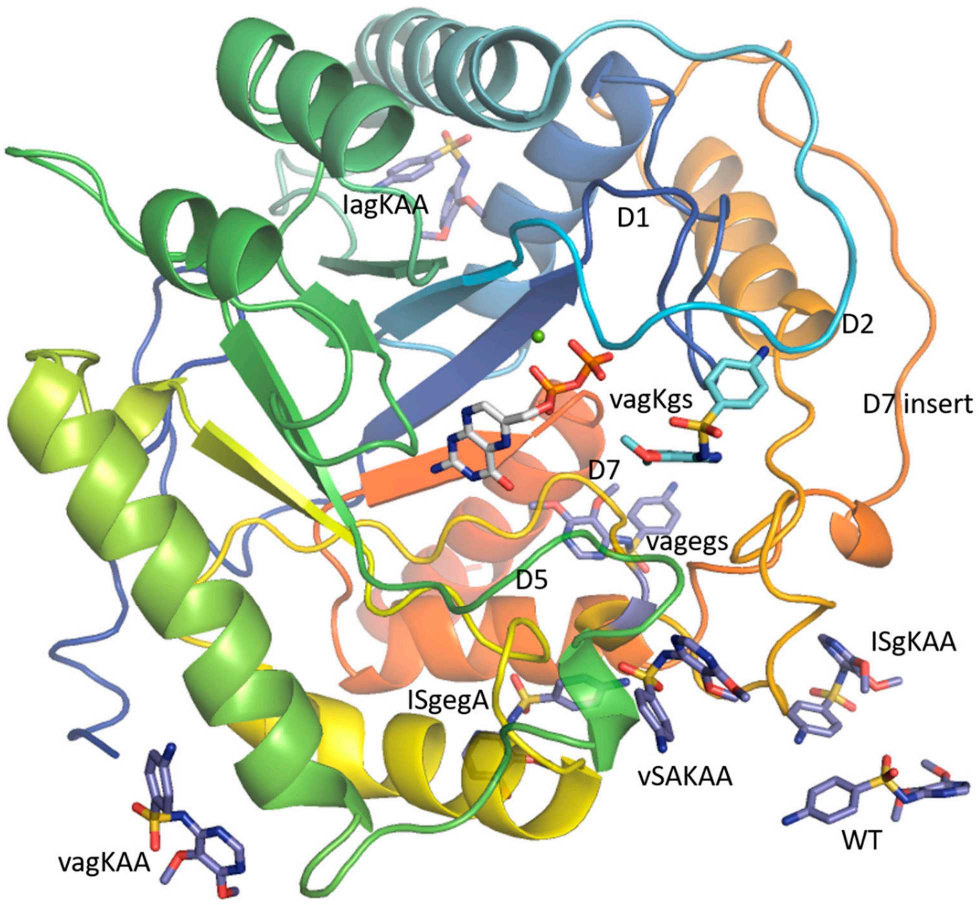

Figure S4. Position of sulfadoxine after unbinding. In the vagKgs system, the sulfadoxine migrates to a site close to the active site and remains stably bound for the rest of the simulation. The sulfadoxine in the vagKgs system is shown in cyan, the others in violet. The system names are shown next to the corresponding sulfadoxine.

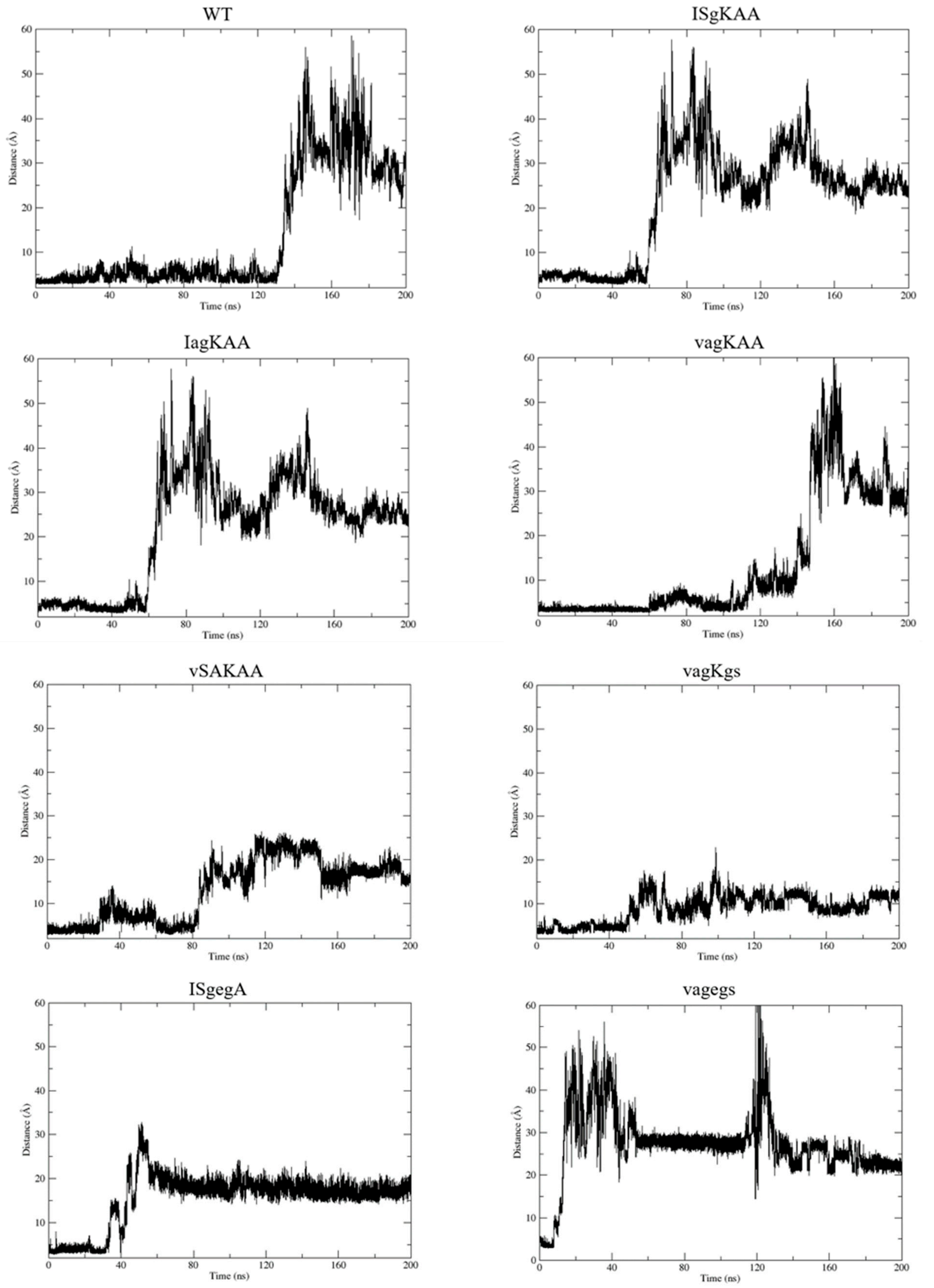

Figure S5. Distance between the C9 atom of 6-hydroxymethyl-7,8-dihydropterin pyrophosphate (DHPPP) and the nitrogen atom of sulfadoxine, measure across all systems over 200 ns of molecular dynamics simulations.

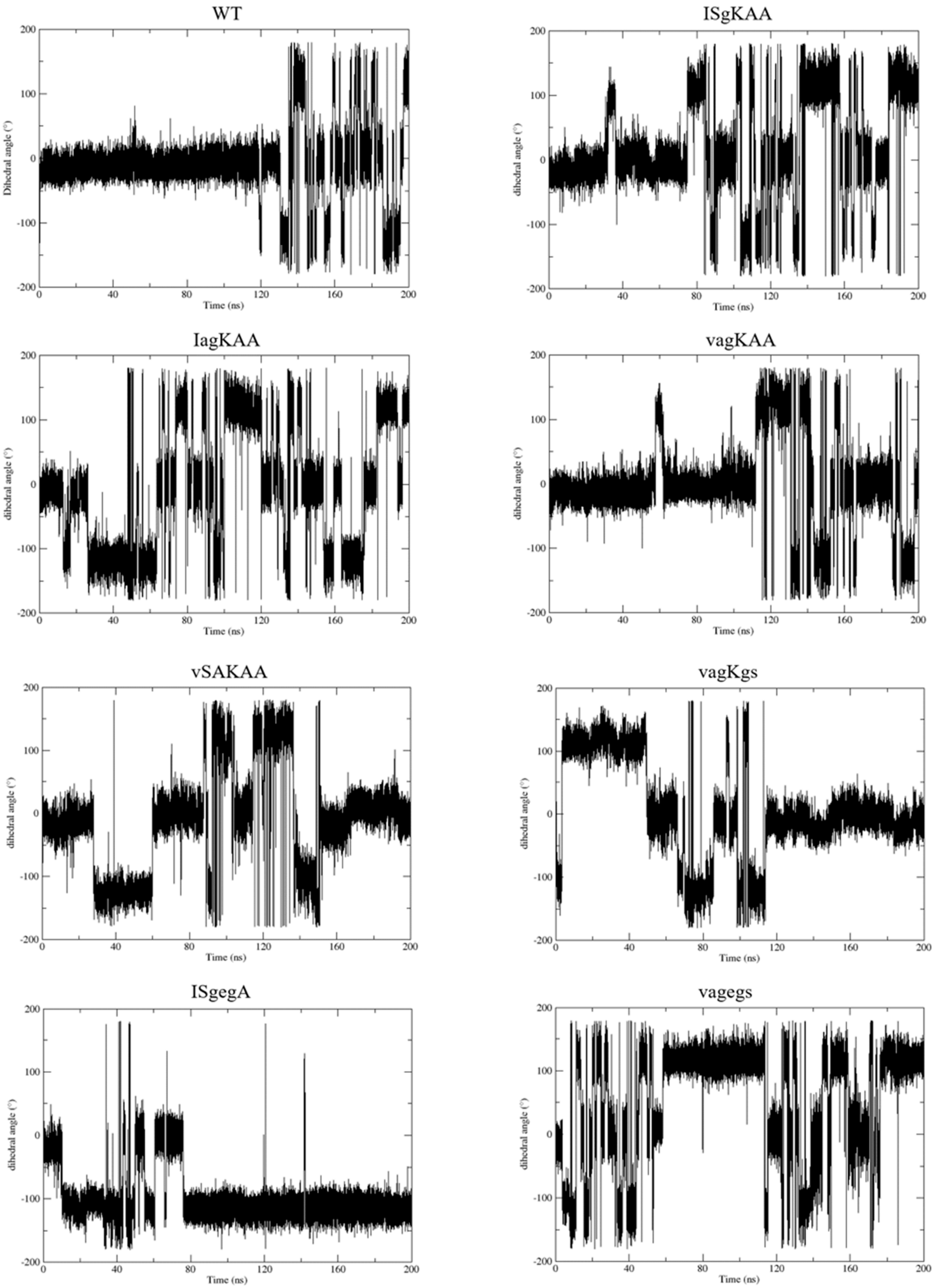

Figure S6. Dihedral angle between atoms C18-S21-N22-C25 of sulfadoxine, measured for each system over 200 ns of molecular dynamics simulations. We identify three different states, with dihedral angle values around -120°, -20° and 130°. This figure should be interpreted alongside Figure S6, as it reveals that conformational changes in sulfadoxine correlate with its dissociation from the active site.

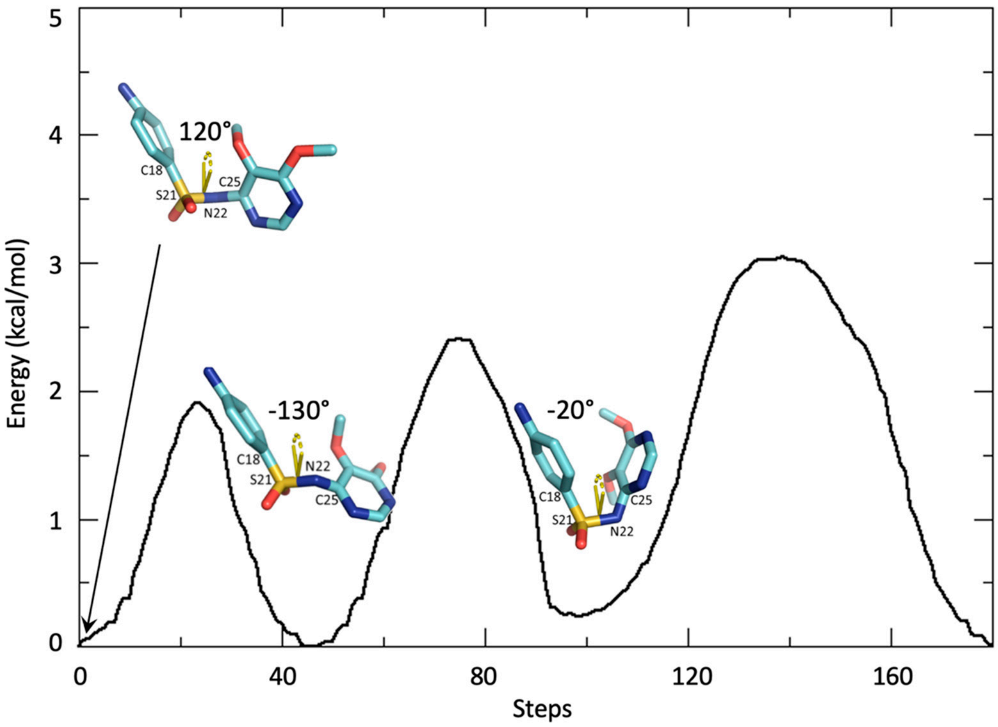

Figure S7. Energy profile for the scan of the C18-S21-N22-C25 dihedral angle in sulfadoxine obtained using the semi-empirical tight binding method GFN2-xTB. The geometry of sulfadoxine was first optimized in implicit solvent, and subsequently used as a starting structure for a dihedral scan performed over 360°, with 180 steps. Three energy minima are identified, separated by low energy barriers of approximately 2-3 kcal/mol. These minima correspond well with the conformations observed throughout the trajectories of molecular dynamics.

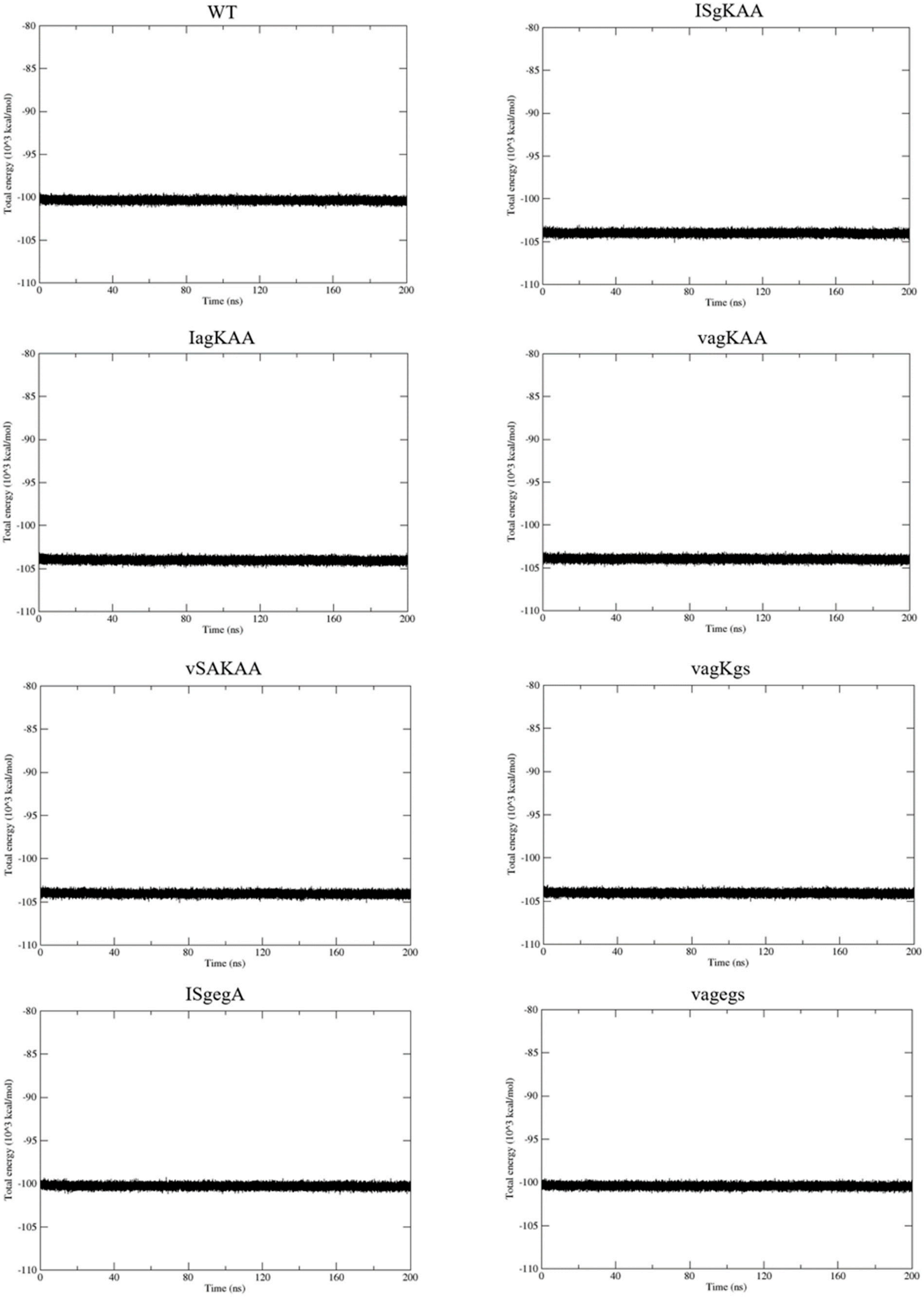

Figure S8. Total energy profiles of the eight *Pf*DHPS systems in the presence of sulfadoxine over 200 ns of molecular dynamics simulations.

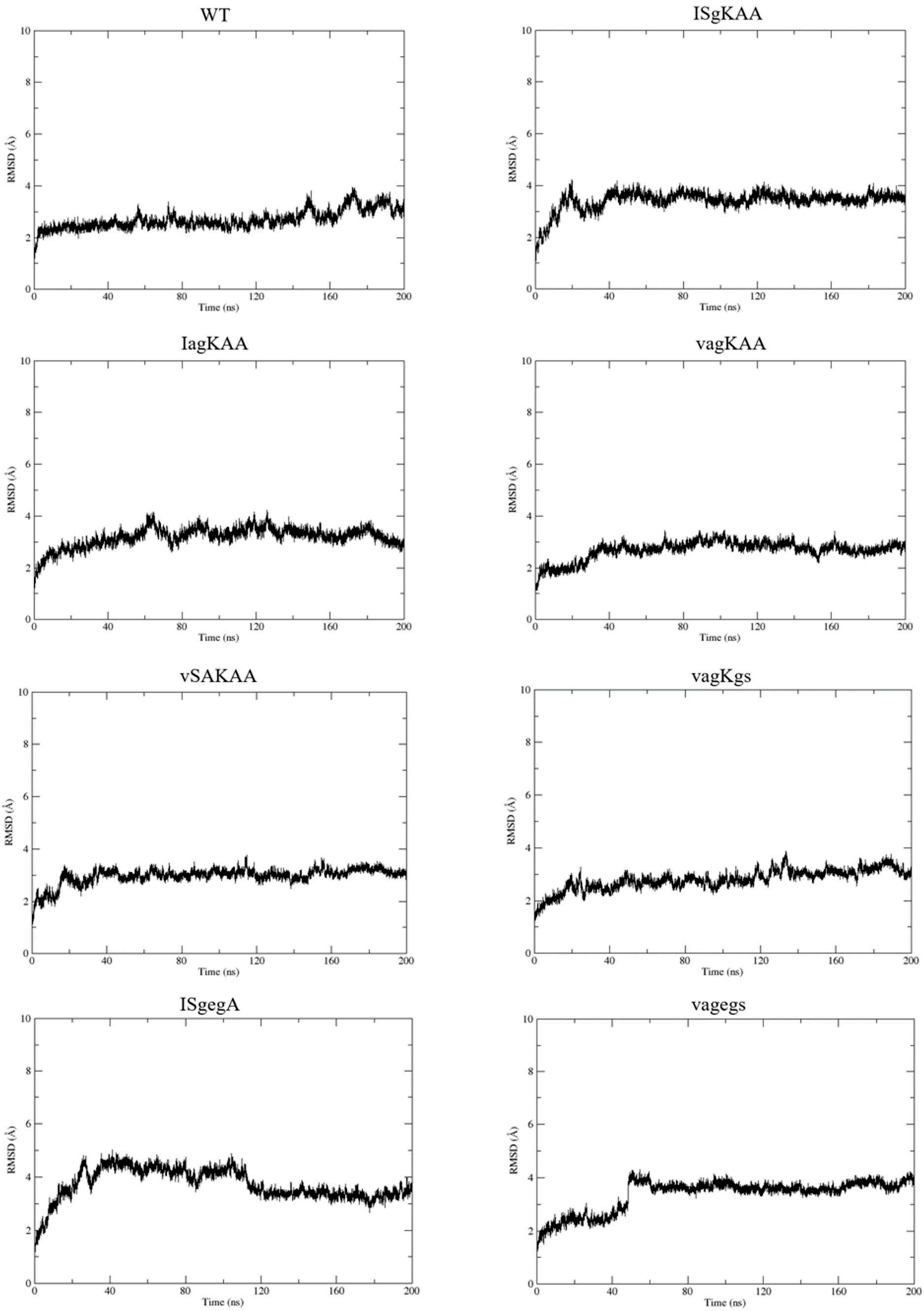

Figure S9. RMSD profiles of all *Pf*DHPS residues across the eight systems in the presence of sulfadoxine over 200 ns of molecular dynamics simulations.

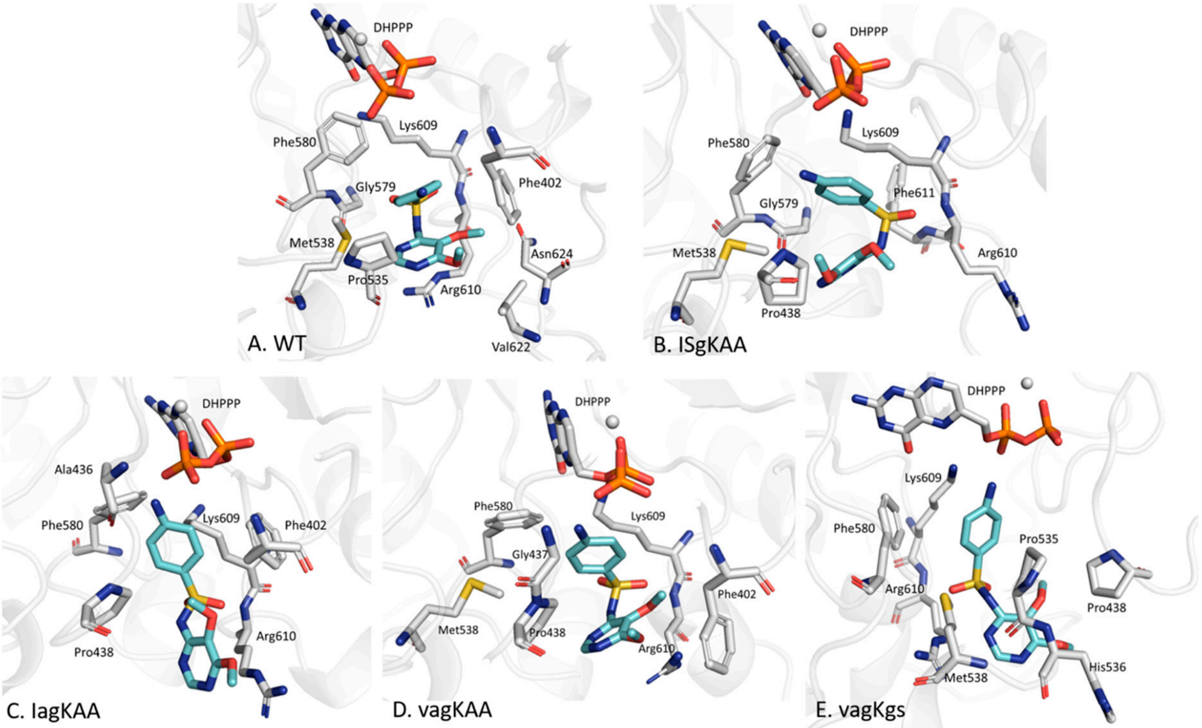

Figure S10. Residues involved in the interaction with sulfadoxine for *Pf*DHPS variants: (A) WT, (B) ISgKAA, (C) IagKAA, (D) vagKAA, and (E) vagKgs. Sulfadoxine is shown in blue. The three key residues conserved across systems are Phe580 (D6), Lys609 and Arg610 (D7).

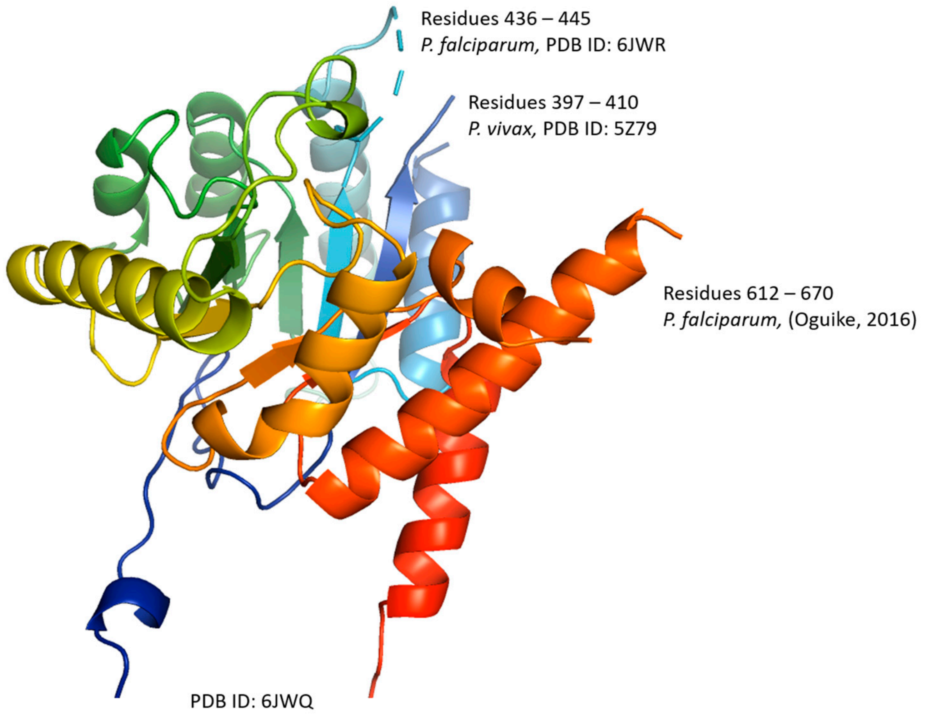

Figure S11. Description of *Pf*DHPS structure building. The initial structure of the WT *Pf*DHPS protein (residues from Ile 366 to Asp 708) was extracted from the Protein Data Bank (PDB) entry 6JWQ. To complete the missing parts, the positions of residues 397 – 410, 436 – 445 and 612 – 670 were extracted from the PDB entries 5Z79 (*Plasmodium vivax*), 6JWR (*Plasmodium falciparum*) and from a homology model, respectively.

Table S1. List of the studied systems with the corresponding mutations of interest.

Codon	I431	S436	A437	K540	A581	A613
**WT (ISAKAA)**	WT	WT	WT	WT	WT	WT
**ISgKAA**	WT	WT	**M**	WT	WT	WT
**IagKAA**	WT	**M**	**M**	WT	WT	WT
**vSAKAA**	**M**	WT	WT	WT	WT	WT
**vagKAA**	**M**	**M**	**M**	WT	WT	WT
**vagKgs**	**M**	**M**	**M**	WT	**M**	**M**
**ISgegA**	WT	WT	**M**	**M**	**M**	WT
**vagegs**	**M**	**M**	**M**	**M**	**M**	**M**

WT: Wild-type; M: mutated; mutations are: isoleucine 431 with valine (I431V), serine 436 with alanine (S436A), alanine 437 with glycine (A437G), Lysine 540 with glutamine (K540E), alanine 581 with glycine (A581G), and alanine 613 with serine (A613S).

Table S2. Residues involved in the *p*ABA pocket. Analysis was performed on the cluster representative frame using IGMPlot and confirmed by using the native contacts command in CPPTRAJ (meaning that interactions are present for at least 50% of the simulation time).

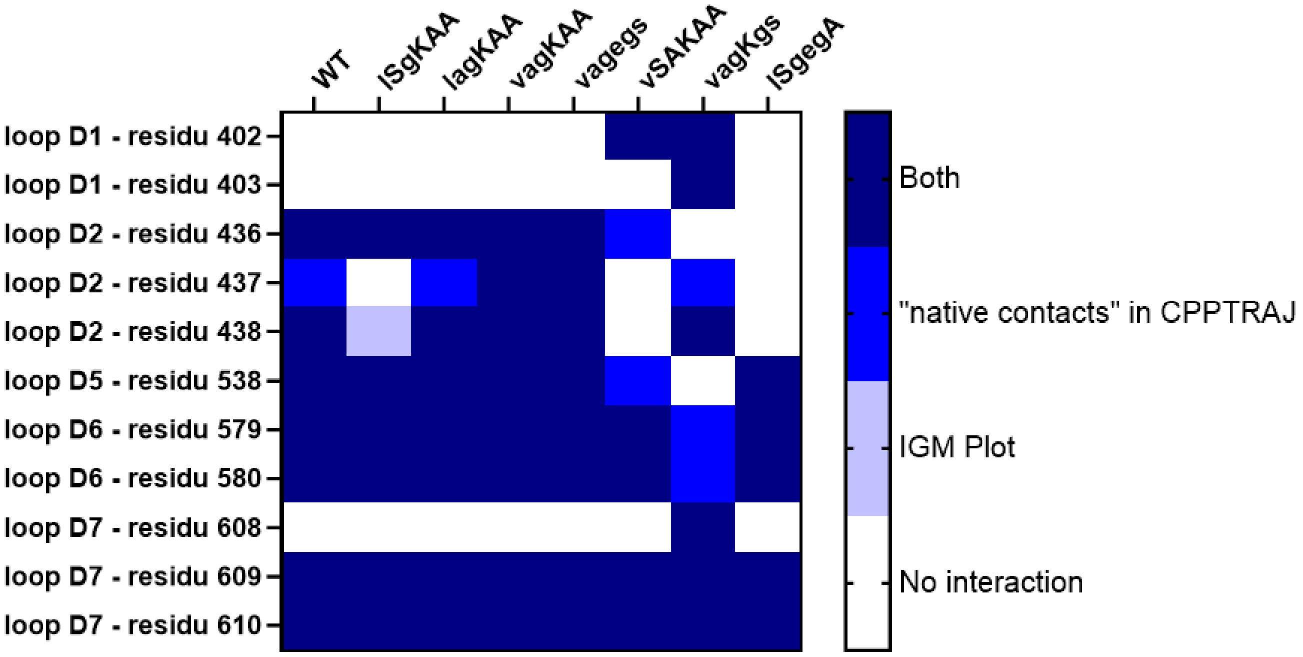

Table S3. Residues involved in the interaction of *Pf*DHPS with DHPPP in systems with natural ligands. Analysis was performed on the cluster representative frame using IGMPlot and confirmed by using the *native contacts* command in CPPTRAJ (meaning that interactions are present for at least 50% of the simulation time).

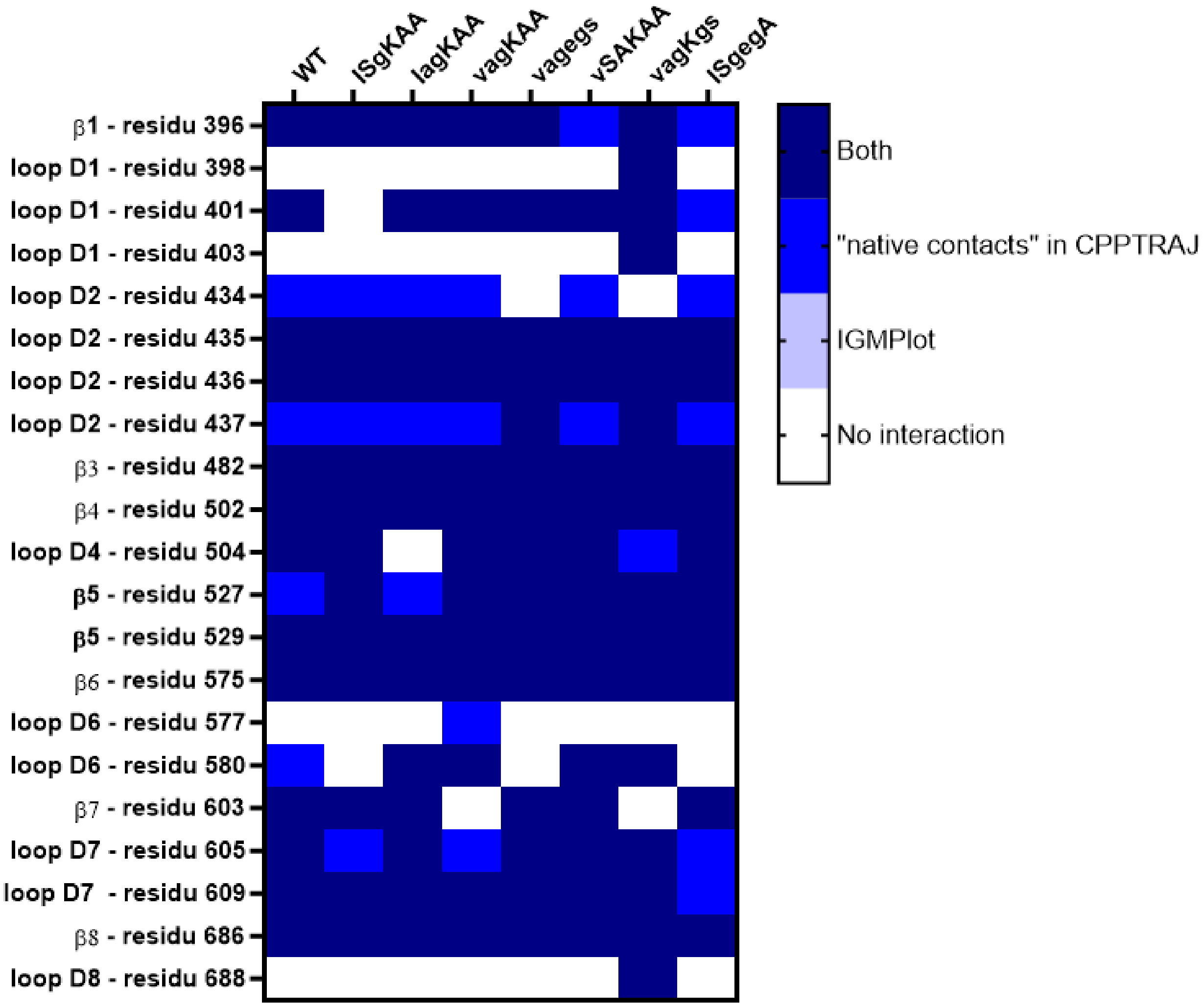

Table S4. Residues involved in the interaction of *Pf*DHPS with DHPPP in the WT systems. Analysis was performed on the cluster representative frame using IGMPlot and confirmed by using the *native contacts* command in CPPTRAJ (meaning that interactions are present for at least 50% of the simulation time).

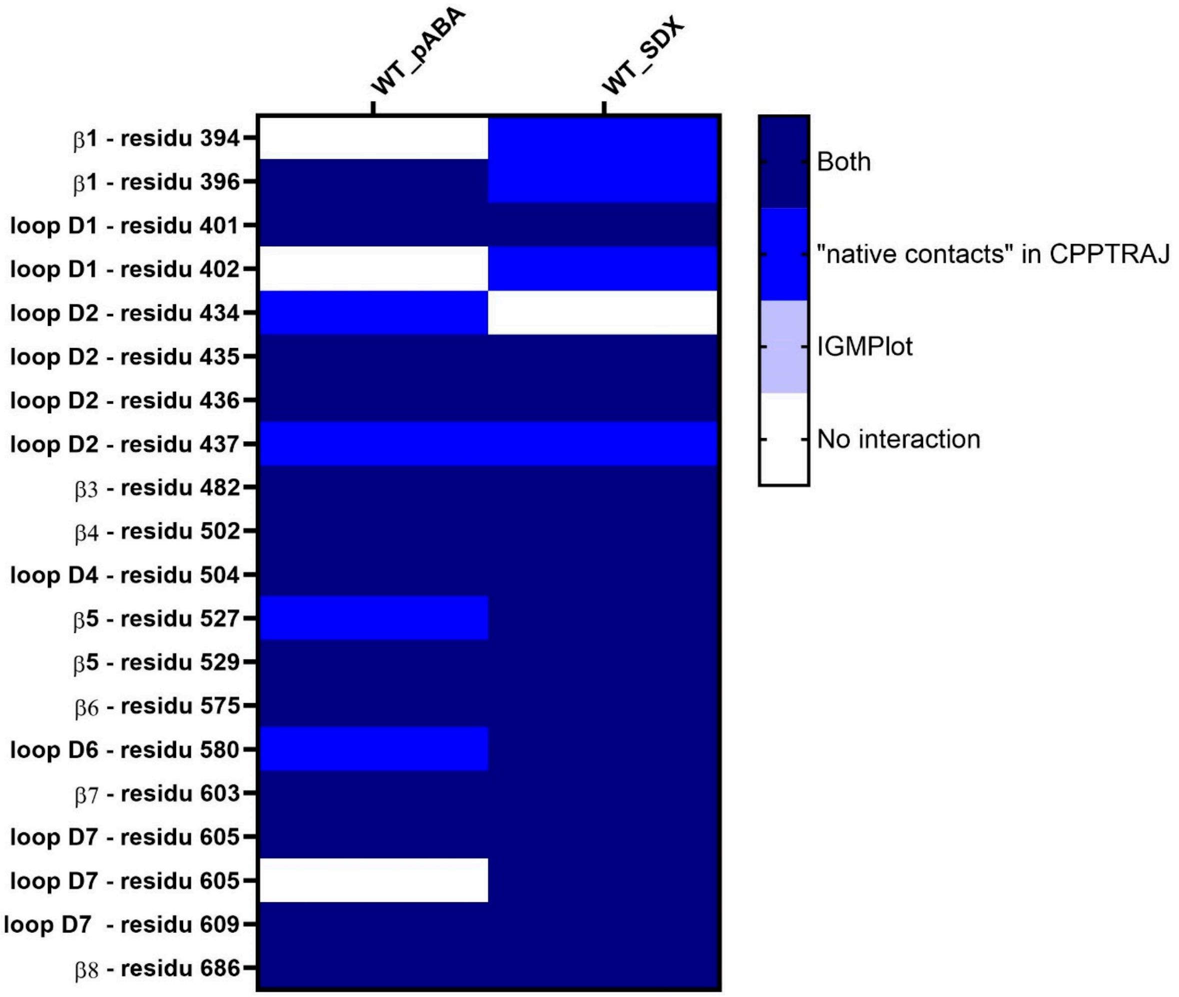

Table S5. Residues involved in the interaction of *Pf*DHPS with *p*ABA or sulfadoxine in the WT systems. Analysis was performed on the cluster representative frame using IGMPlot and confirmed by using the *native contacts* command in CPPTRAJ (meaning that interactions are present for at least 50% of the simulation time).

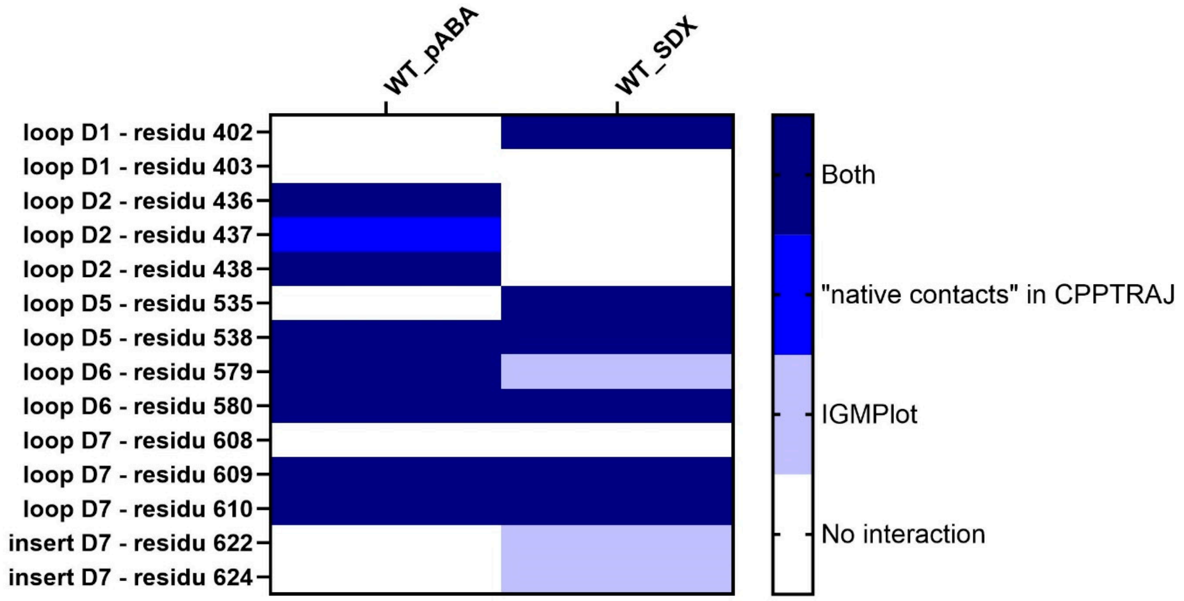

Table S6. Residues involved in the interaction of *Pf*DHPS with sulfadoxine. Analysis was performed on the cluster representative frame using IGMPlot and confirmed by using the *native contacts* command in CPPTRAJ (meaning that interactions are present for at least 50% of the simulation time).

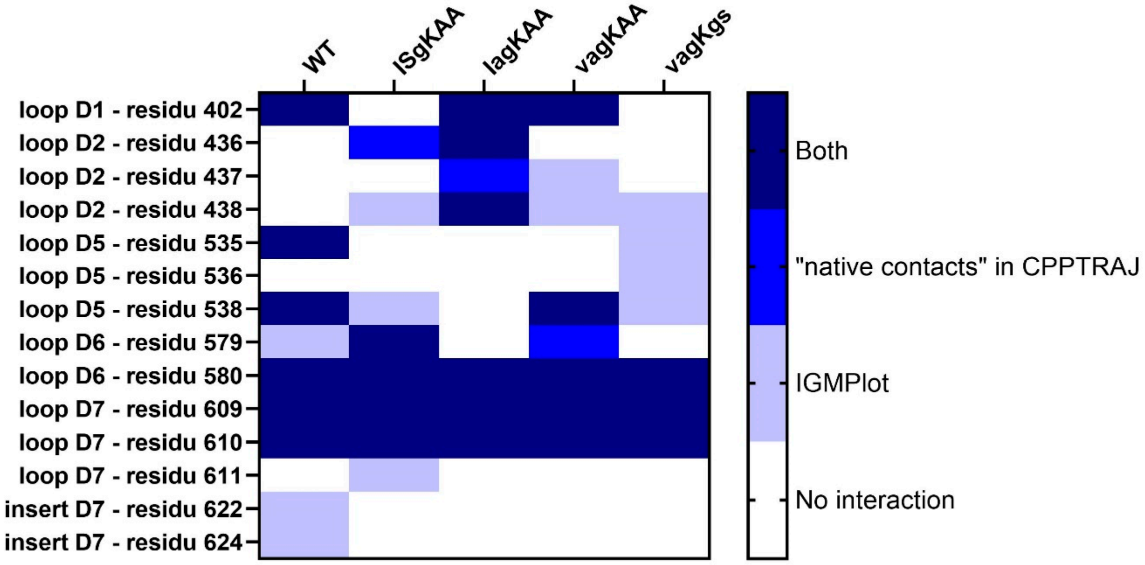

Supplement: Supplementary file 1 [file molecules-30-04118-s001.zip › molecules-3871073-supplementary.pdf]
